# Hierarchical and Fractal-Inspired Mechanical Metamaterials: A Mechanics-Oriented Review of Multiscale Design, Strength, Deformation, and Energy Absorption

**DOI:** 10.3390/ma19163432

**Published:** 2026-08-13

**Authors:** Saulius Diliūnas, Vitalis Leišis, Tilmutė Pilkaitė, Inga Skiedraitė

**Affiliations:** Faculty of Mechanical Engineering and Design, Kaunas University of Technology, 51424 Kaunas, Lithuania; vitalis.leisis@ktu.lt (V.L.); tilmute.pilkaite@ktu.lt (T.P.); inga.skiedraite@ktu.lt (I.S.)

**Keywords:** mechanical metamaterials, hierarchical structures, fractal-inspired architectures, multiscale design, failure modes, strength-to-weight performance, energy absorption, crashworthiness, architecture-controlled mechanics

## Abstract

Classical continuum descriptions based on homogenized parameters, such as modulus and relative density, become inadequate when performance is governed by deliberately engineered multiscale architecture rather than by composition, as in hierarchical and fractal-inspired mechanical metamaterials. In these materials, geometry couples deformation across length scales in ways that single-scale models cannot capture. This review synthesizes theoretical, numerical, and experimental literature published mainly over the last two decades, with emphasis on the last five years. It draws preferentially on peer-reviewed journal articles and highly cited studies; strictly periodic single-scale metamaterials and general electromagnetic–metamaterial literature are cited only where needed to establish historical context and are not otherwise within the scope of this review. The specific contribution of this review is threefold: (i) a mechanics-oriented classification of hierarchical and fractal-inspired architectures according to the deformation mechanism they activate (bending-, stretching-, membrane-, or buckling-dominated), rather than by geometric appearance alone; (ii) an explicit structure-mechanism-property-function framework linking multiscale geometry to strength, failure evolution, and energy absorption, tested against automotive crashworthiness as an application case study; and (iii) a critical account of when fractal–mathematical descriptors (scale invariance, similarity ratio, iteration depth, and fractal dimension) are mechanically meaningful, since many finite hierarchical lattices are fractal-inspired rather than fractal in the strict mathematical sense. The synthesis shows that relative density alone is an insufficient performance descriptor: outcomes depend on whether deformation is bending-, stretching-, membrane-, or buckling-dominated, and hierarchy or fractal-inspired recursion improves performance only when each structural level is assigned a distinct mechanical role rather than repeating geometry without function. The crashworthiness case study confirms that no single architecture is universally optimal once force efficiency, intrusion control, and manufacturability are considered alongside energy absorption. Future progress requires integrated structure-mechanism-property-function frameworks that combine theoretical modeling, high-resolution simulation, data-driven design, and experimental validation, with complexity justified only when it is mechanically purposeful, validated, and manufacturable at scale.

## 1. Introduction

The mechanical behavior of solids has traditionally been described within continuum mechanics, where material response is represented by a limited set of effective parameters, such as elastic moduli, Poisson’s ratio, and strength measures. This description assumes that the internal structure of a material is either homogeneous or sufficiently regular for its influence to be averaged through homogenization. Although these assumptions remain adequate for many conventional engineering materials, they become increasingly restrictive as internal architectures become more complex, heterogeneous, and deliberately engineered.

Even when materials are produced from the same base constituent, differences in internal architecture can produce fundamentally distinct mechanical responses. A monolithic elastomer and a porous elastomer derived from the same polymer may exhibit markedly different elastic stiffness, shear response, Poisson’s ratio, and failure behavior. These differences do not arise from changes in intrinsic material chemistry, but from how stresses and strains are redistributed through the internal architecture. In porous and cellular systems, this redistribution is governed by pore size, shape, connectivity, and spatial arrangement, which together define the effective mechanical performance of the material.

Functionally graded porous materials extend this concept by allowing porosity to vary gradually from the surface toward the interior. In such systems, each internal layer possesses different local stiffness and strength, while the macroscopic response emerges from mechanical interactions across layers. Although this strategy offers additional freedom in tailoring performance, it remains constrained by the relatively simple geometric nature of conventional pore-based architectures. Consequently, the range of attainable combinations of stiffness, strength, and deformation behavior remains limited, and the resulting stress fields can often still be interpreted within the framework of classical homogenized descriptions.

In recent years, the search for advanced material solutions has been increasingly driven by engineering sectors such as automotive or aircraft design, where performance requirements extend far beyond conventional strength and stiffness. Modern vehicle structures demand lightweight construction, high energy efficiency, crashworthiness, vibration attenuation, long-term durability, and compatibility with sustainability-oriented engineering goals. Materials that reduce mass without compromising structural integrity or functional reliability are therefore of central importance in next-generation mobility systems. Within this context, architected materials and metamaterials have emerged as a particularly promising class of material systems. In particular, hierarchical and fractal metamaterial architectures offer new opportunities for the development of lightweight, high-performance, and multifunctional composite structures for vehicles, energy-absorbing components, and other demanding structural applications. Their ability to tailor mechanical response across multiple length scales makes them especially attractive for future automotive materials and other advanced engineering systems.

Architected metamaterials challenge the limitations of conventional materials by demonstrating that mechanical properties can be designed primarily through geometry rather than composition. Through the use of periodic or non-periodic structural motifs, metamaterials have been shown to exhibit non-classical responses such as negative Poisson’s ratio, negative stiffness, extreme anisotropy, and programmable deformation pathways. However, many existing metamaterial designs rely on periodic unit cells characterized by a single dominant length scale, which inherently limits the range of mechanical interactions that can develop within the structure.

Hierarchical and fractal-inspired architectures represent a qualitatively different design paradigm. Strict mathematical fractals are defined by self-similar geometries that repeat over multiple length scales and by non-integer fractal dimensions that quantify geometrical complexity. Engineered architectures, however, are finite and manufacturing-limited; therefore, many structures described in the mechanical-metamaterials literature are more accurately termed fractal-inspired rather than fractal in the strict mathematical sense. Their relevance lies in the ability to couple mechanical behavior across scales, allowing local deformations at small scales to influence the global response and vice versa.

From a theoretical standpoint, such materials expose the limitations of classical continuum descriptions. In hierarchical and fractal structures, stress and strain fields are highly heterogeneous and frequently exhibit localization, scale dependence, and non-affine deformation modes. Effective material parameters alone are therefore insufficient to describe these responses, because they obscure the internal stress pathways that govern stiffness, strength, stability, and failure. Consequently, understanding the mechanical performance of fractal and hierarchical metamaterials requires explicit analysis of internal stress–strain distributions and their evolution under different loading conditions.

This issue becomes especially important when strength and failure are considered. Although unconventional effective properties such as negative Poisson’s ratio or negative stiffness have attracted considerable attention, the practical application of such materials ultimately depends on load-bearing capacity, damage tolerance, and failure mechanisms. In hierarchical architectures, these properties are controlled by the manner in which stresses concentrate or redistribute across scales, how local instabilities interact with global deformation, and how damage initiates and propagates within the internal architecture. Without a clear understanding of these processes, the rational design and reliable deployment of fractal-based materials remain limited.

A meaningful assessment of these issues requires the integration of theoretical, numerical, and experimental evidence. Although research on metamaterials and fractal structures has expanded rapidly across mechanics, materials science, and applied physics, the literature remains fragmented, with different research communities emphasizing different aspects of geometry, scale, functionality, and application. A comprehensive review is therefore needed to identify common trends, methodological strategies, and unresolved questions regarding the mechanical behavior of hierarchical and fractal-inspired architectures.

The purpose of this article is to review the current state of research on hierarchical and fractal-inspired mechanical metamaterials from a mechanics-oriented perspective, with particular emphasis on how internal architecture governs stress distribution, deformation behavior, and strength. By synthesizing existing approaches and identifying key gaps, this review aims to clarify how research on complex architected materials is evolving and to provide a mechanics-based foundation for future studies.

Consistent with it, the literature base for this review was assembled from peer-reviewed journal articles, with priority given to studies published within the last five years and to foundational, highly cited works from the last two decades where they remain the primary reference for a given concept (e.g., cellular-solid scaling laws, the octet-truss bound, or the original demonstrations of negative-index metamaterials). Conference proceedings and non-peer-reviewed sources were avoided except where no equivalent peer-reviewed source existed. Electromagnetic metamaterials and fractal antennas are discussed only in Section 2, to the extent necessary to explain the historical and terminological origin of the field; they are not treated as part of the mechanics-oriented synthesis that follows.

## 2. Historical Development of Fractal Structures and Metamaterials

The origins of fractal structures and metamaterials emerged from distinct scientific disciplines and evolved largely independently before converging into the modern field of fractal-inspired metamaterials. The mathematical foundations of fractal geometry were established through the work of Mandelbrot, who introduced the term fractal and demonstrated the relevance of self-similar geometries for describing complex natural forms and irregular surfaces [1,2]. During the 1980s, fractal concepts were increasingly applied to characterize porous media, fracture surfaces, catalysts, and other heterogeneous materials, providing a powerful framework for describing multiscale geometrical complexity rather than for designing engineered structures.

An important transition from analysis to engineering occurred in the 1990s with the development of fractal antennas. Self-similar geometries such as the Sierpiński gasket and Koch curve enabled antenna miniaturization and multi-band electromagnetic performance, representing one of the first intentional technological applications of fractal design principles [3,4]. At approximately the same time, a separate research direction was emerging in electromagnetics.

The theoretical foundations of metamaterials can be traced to the seminal work of Veselago, who predicted the unusual electromagnetic behavior of materials possessing simultaneously negative permittivity and permeability [3,5,6]. However, practical realization became possible only after the pioneering studies of Pendry and co-workers on artificial electromagnetic structures, including metallic wire arrays [7] and split-ring resonators [3,8]. These developments culminated in the first experimental demonstration of a negative-index metamaterial by Smith et al. in 2000 [5,9], marking the beginning of the modern metamaterial era.

During the following decade, metamaterial concepts rapidly expanded beyond electromagnetics into acoustics, elastodynamics, and mechanics. Simultaneously, advances in additive manufacturing enabled the fabrication of increasingly complex hierarchical architectures. Consequently, research attention shifted from wave manipulation toward architected materials with tailored mechanical responses, including auxetic, pentamode, and lattice-based metamaterials [1,10,11,12,13,14,15].

The independent emergence of these two research directions, followed by their convergence around the year 2000, highlights the potential of interdisciplinary knowledge transfer. This convergence shows how concepts initially developed in electromagnetics, acoustics, and antenna engineering can stimulate advances in mechanics and materials science. In this context, the adaptation of principles from adjacent scientific domains represents an important driver of innovation in modern materials research and engineering.

**Figure 1:**
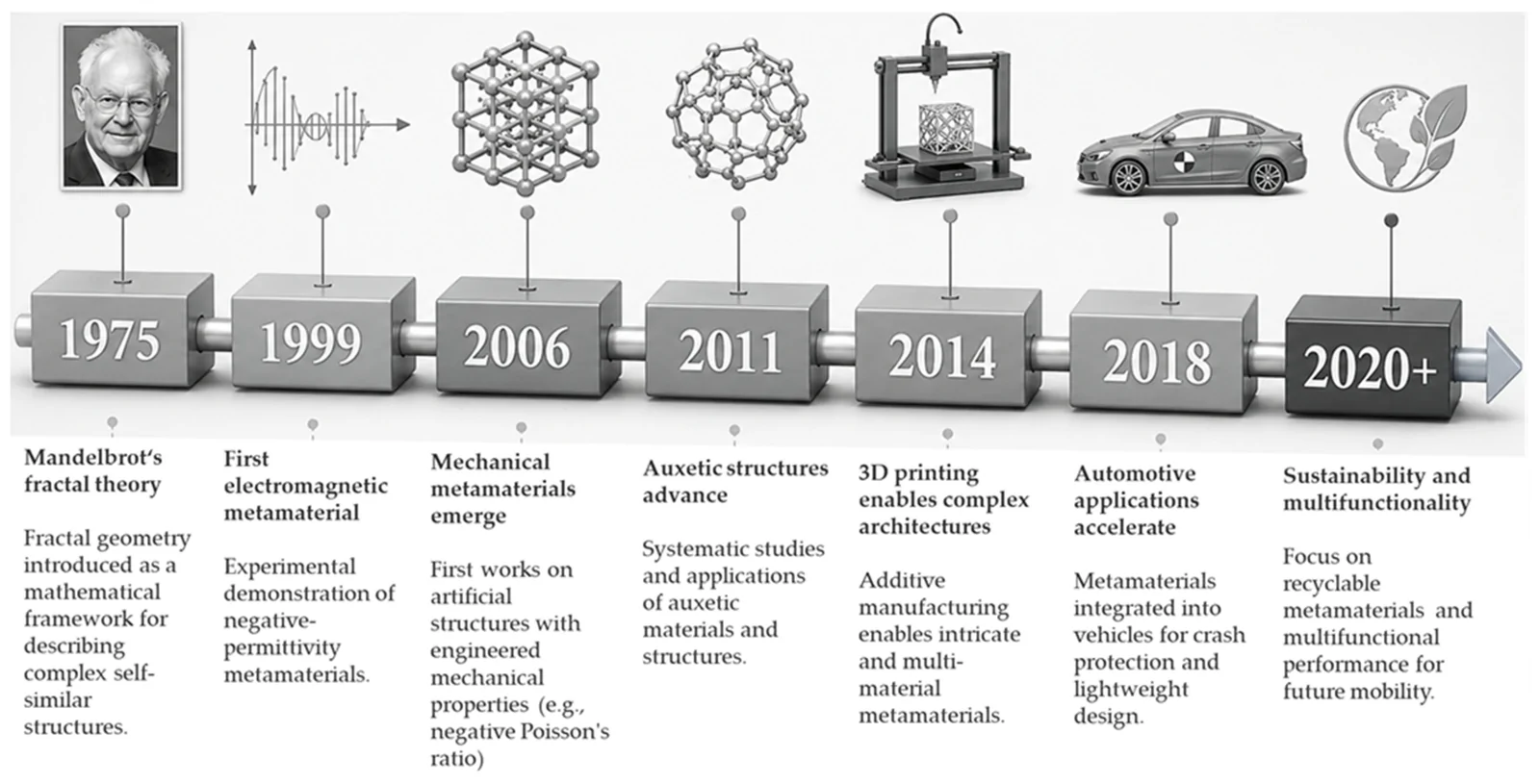
Evolution of fractal geometry and mechanical metamaterials. Schematic timeline compiled from representative foundational and review publications [1,2,3,4,5,6,7,8,9,10,11,12,13,14,15]. The indicated years denote approximate periods of major developments rather than exact publication dates. The figure was created with assistance from ChatGPT (OpenAI) GPT-5.6 Luna and subsequently reviewed, edited, and scientifically validated by the authors.

Since approximately 2015 (Figure 1), fractal geometries and metamaterial design philosophies have increasingly converged. Hierarchical and self-similar architectures have been incorporated into mechanical metamaterials to enhance stiffness-to-weight ratio, energy absorption, damage tolerance, and multifunctionality. This convergence has established fractal mechanical metamaterials as a rapidly growing research field, driven by advances in computational design, topology optimization, and additive manufacturing technologies.

This qualitative trend is reflected in the literature discussed in the following sections. Foundational studies on hierarchical lattices and triply periodic minimal surface architectures discussed in Section 3 are concentrated mainly in the 2000s and early 2010s [16,17,18,19,20]. The studies on crashworthiness, vibration attenuation, nanolattices, and data-driven design discussed in Section 3, Section 4, Section 5, Section 6 and Section 7 are predominantly dated 2020–2025 [21,22,23,24,25,26,27,28,29,30,31,32]. This distribution supports the view that experimental and application-oriented research on fractal-inspired mechanical metamaterials has accelerated markedly in recent years.

## 3. Architecture-Governed Deformation and Failure: Beyond Relative Density

### 3.1. Beyond Relative Density: Architecture as the Primary Design Variable

The mechanical performance of modern metamaterials should not be interpreted solely through the intrinsic properties of their constituent materials. Although elastic modulus, strength, toughness, and density remain essential descriptors, they are insufficient when the dominant mechanical response is governed by architecture, topology, characteristic length scale, and deformation mode [16,33,34,35,36]. The central contribution of architected metamaterials is therefore not merely mass reduction, but the deliberate organization of matter to redirect load paths, control instability, delay damage localization, and tune energy dissipation [16,34,35,36]. This represents a substantial departure from conventional porous solids, in which decreasing density usually causes a pronounced deterioration in stiffness and strength because deformation is dominated by bending of randomly arranged cell walls or struts [16,37]. In contrast, well-designed metamaterials aim to preserve structural efficiency by promoting axial stretching, membrane stresses, reversible elastic buckling, progressive collapse, or wave attenuation [16,21,22,33,35,38]. The critical issue is thus not whether a material is porous or lightweight, but whether the remaining solid phase is arranged so that it performs a mechanically useful function [16,33,34,35,36].

This distinction is important because many reported advances in mechanical metamaterials can be understood as attempts to overcome the limitations of classical cellular solids [16,34,35,36]. In stochastic foams, load transfer is inefficient because the internal architecture is not optimized [16,37]. The material may contain a large number of load-bearing elements, but many of them deform by bending, rotate at junctions, or contribute only weakly to global stiffness [16,33]. Architected metamaterials address this problem by replacing random porosity with geometrically programmed load-bearing networks [16,34,36]. However, ordered architectures are not necessarily mechanically superior [33,35]. Periodicity, symmetry, or visual complexity alone do not guarantee high stiffness, strength, recoverability, or damage tolerance [17,33,35]. A critical evaluation should therefore focus on the deformation mechanisms activated by a given architecture. The same relative density can produce markedly different mechanical responses depending on whether the structure deforms through bending, stretching, shell buckling, membrane action, local fracture, or hierarchical energy dissipation [17,21,33,35].

Relative-density scaling provides a useful framework for comparing these mechanisms [16,33,37]. Classical cellular solids often follow steep power–law relationships between relative density and mechanical properties, particularly when bending dominates. In such cases, even modest density reduction can cause a disproportionate loss of stiffness and strength. Stretch-dominated lattices, such as octet-truss architectures, were introduced as a response to this limitation. Their high connectivity allows structural members to carry loads primarily in tension or compression, leading to more favorable stiffness–density scaling than that of bending-dominated foams. The significance of such architectures is not simply that they achieve high specific stiffness, but that they demonstrate a fundamental principle: the mechanical penalty associated with low density is not unavoidable. It is largely a consequence of inefficient load transfer. By suppressing bending and promoting axial force transmission, lightweight materials can retain a much larger fraction of their load-bearing capability [16,39]. A concrete illustration of geometry-controlled load transfer is provided by TPMS-based fillets at the nodes of metal cubic lattices, which modify the local stress field and induce a favorable triaxial stress state, thereby improving the strength-to-weight ratio under both static and dynamic loading relative to fillet-free lattices [28]. The example supports the broader point that internal architecture governs load transfer, stress concentration, and failure resistance, and is not merely a matter of visual complexity.

Nevertheless, stretch-dominated truss lattices should not be regarded as the ultimate solution for lightweight mechanical design [33]. Their performance depends strongly on idealized assumptions, including accurate member alignment, robust node geometry, uniform cross-sections, and well-defined boundary conditions [22,35]. In practical structures, these assumptions are often violated [35,40]. Nodes may act as stress concentrators, slender members are susceptible to buckling, and manufacturing imperfections can redirect deformation away from the intended axial load path [33,35,40]. Moreover, even highly optimized strut-based architectures remain fundamentally constrained by one-dimensional load transfer through discrete members, which may limit the uniform distribution of stresses and strain energy under complex loading conditions. Theoretical and computational studies have consequently suggested that plate- and shell-based cellular architectures can approach the Hashin–Shtrikman upper bounds for isotropic elastic stiffness more closely than conventional truss lattices because continuous two-dimensional surfaces support membrane stresses more efficiently than discrete struts [33]. Therefore, the superior performance of stretch-dominated lattices should not be interpreted as universally optimal; rather, structural efficiency is governed by the compatibility between architecture, topology, geometry, and the dominant deformation mechanism [16,33].

**Figure 2:**
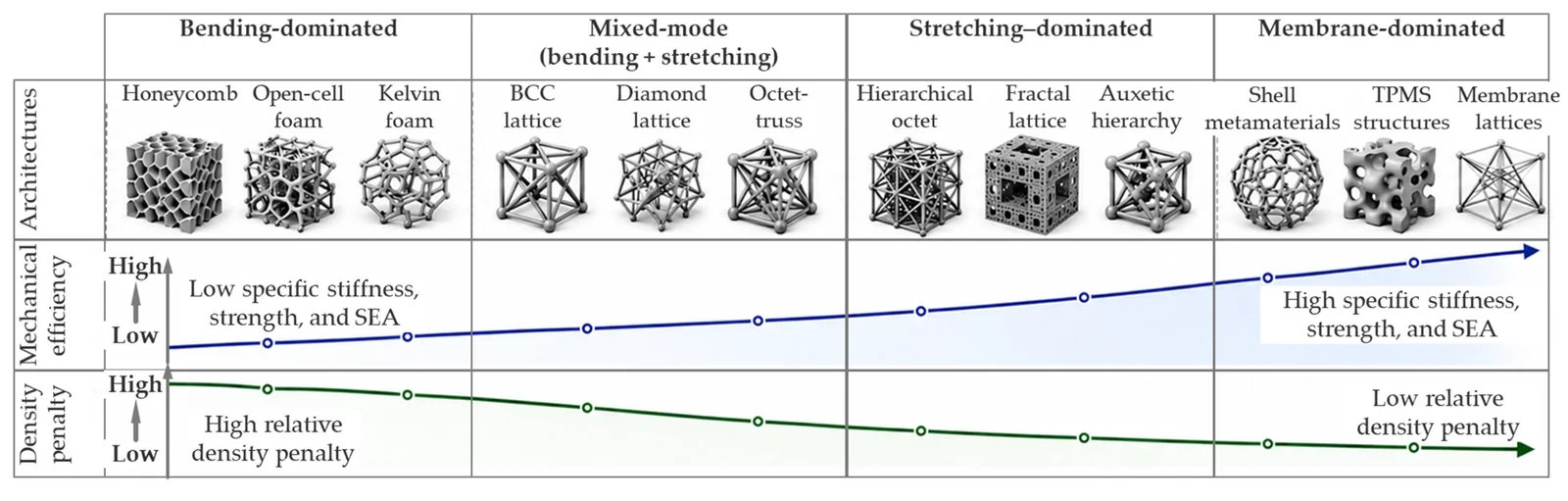
Conceptual overview of structure–mechanism–property relationships in architected metamaterials: general transition from bending-dominated to stretching-dominated deformation regimes and the associated influence of topology, relative density, and hierarchical organization on structural efficiency. Generalized framework derived from scaling arguments and representative geometrical concepts reported in the literature [16,33,37,39,41,42]. The figure was created with assistance from ChatGPT (OpenAI) GPT-5.6 Luna and subsequently reviewed, edited, and scientifically validated by the authors.

This progressive relationship is summarized schematically in Figure 2, which organizes representative cellular architectures along a continuum of load-transfer mechanisms extending from bending- to membrane-dominated deformation. At the bending-dominated end, conventional honeycombs, open-cell foams, and Kelvin foams derive their mechanical response primarily from bending of cell walls or edges, resulting in relatively low specific stiffness, strength, and specific energy absorption (SEA), together with a pronounced density penalty that arises from inefficient material utilization. Intermediate architectures, including body-centered cubic (BCC) lattices, diamond lattices, and octet trusses, increasingly activate axial deformation while still retaining a mixed bending–stretching response, thereby progressively improving structural efficiency. Fully stretch-dominated topologies, represented by hierarchical octet lattices, fractal lattice architectures, and hierarchical auxetic systems, transfer loads predominantly through axial member stretching, enabling substantially higher specific stiffness and strength while reducing the density penalty. Finally, shell metamaterials, triply periodic minimal surface (TPMS) structures, and membrane lattices occupy the membrane-dominated regime, where continuous curved surfaces sustain loads primarily through membrane stresses. This deformation mode provides the most efficient material utilization, combining high specific stiffness, strength, and energy absorption with the lowest relative density penalty. Consequently, the figure illustrates that mechanical performance improves systematically as the dominant deformation mechanism evolves from bending through mixed bending–stretching and stretch-dominated behavior toward membrane-dominated load transfer, emphasizing that deformation mechanism, rather than topology alone, governs the efficiency of lightweight mechanical metamaterials [41,42].

### 3.2. Stretch-Dominated Lattices, Shells, and TPMS Architectures

**Figure 3:**
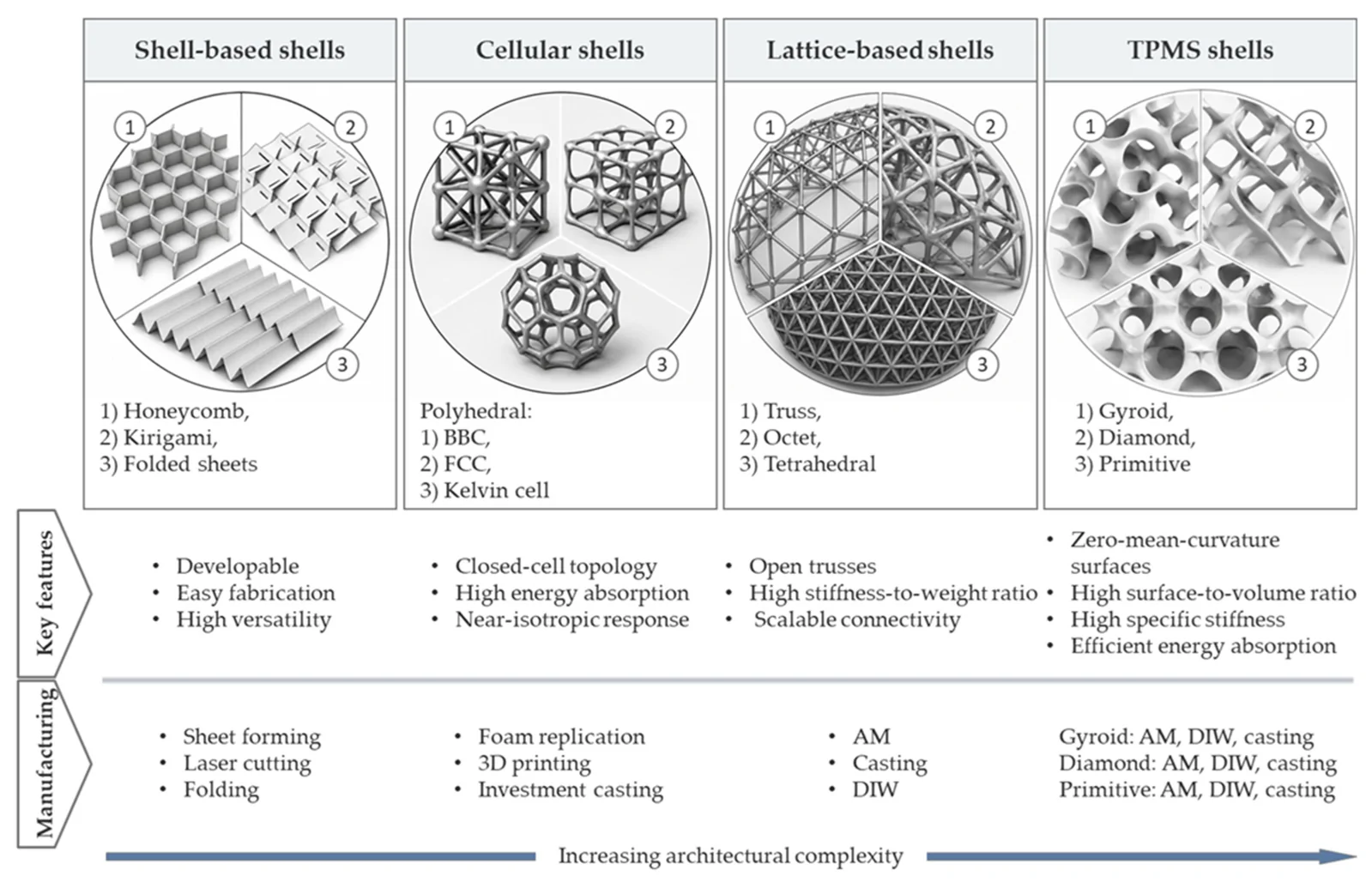
Overview of shell-based mechanical metamaterial architectures. Representative surface-based architectural subclasses, including shell-based cellular solids, lattice-derived shells, and triply periodic minimal surface (TPMS) shells, arranged according to increasing geometric complexity. The TPMS schematics (e.g., gyroid, diamond, primitive, and I-WP) are based on representative studies [18,19,45], whereas the inverse-opal and semi-open shell concepts are adapted from [43,44]. The figure was created with assistance from ChatGPT (OpenAI) GPT-5.6 Luna and subsequently reviewed, edited, and scientifically validated by the authors.

This realization has shifted attention toward plate, shell, and quasi-closed-cell architectures. Such structures can engage a larger fraction of the solid phase in load bearing because stresses are distributed over surfaces rather than concentrated in slender members. From a mechanical perspective, this is highly advantageous: membrane-dominated deformation is generally more efficient than bending-dominated deformation, especially at low relative density. Shell-like structures, inverse opals, and related surface-based geometries therefore offer a promising route toward high stiffness and strength at reduced mass [33,43,44]. Within this surface-dominated category, triply periodic minimal surface (TPMS) architectures—such as gyroid, diamond, primitive, and I-WP topologies—represent a particularly active subclass, combining zero mean curvature with continuous double connectivity. It can provide smooth, imperfection-tolerant load paths and high specific energy absorption (Figure 3) [18,19]. However, their advantages are accompanied by new limitations. Thin shells may be highly efficient before instability, but they can also be sensitive to local buckling, geometric imperfections, shell-thickness variations, and boundary constraints [35,40,44]. A shell architecture that performs exceptionally under ideal compression may become less reliable under multiaxial loading, impact, fatigue, or imperfect manufacturing conditions [35,38,40,44]. Consequently, shell-based metamaterials should not be evaluated only by peak modulus or strength [35,44]. Their post-buckling stability, failure progression, imperfection tolerance, and manufacturability are equally important [35,40,44].

### 3.3. Nanolattices and Ceramic Metamaterials: Size Effects and Failure-Mode Control

Nanolattices add a further dimension to this discussion because they combine architecture-enabled load bearing with size-dependent strengthening. When characteristic dimensions are reduced to the micro- or nanoscale, the probability of critical defects in the load-bearing material can decrease, allowing the constituent solid to approach much higher apparent strengths than in bulk form [22]. This effect is particularly important for brittle or flaw-sensitive materials [21,22,46]. Carbon nanolattices, for example, demonstrate that porosity and high strength need not be mutually exclusive when nanoscale features reduce flaw sensitivity and architecture distributes load effectively. One of the principal contributions of nanolattices is therefore twofold: they reduce mass through porosity while simultaneously exploiting small-scale strengthening of the solid phase. This partial decoupling of strength from density is difficult to achieve in conventional cellular solids [22].

At the same time, nanoscale performance should be interpreted cautiously. Many exceptional mechanical properties reported for nanolattices are measured using very small specimens under controlled loading conditions [22,35]. These experiments are essential for identifying fundamental mechanisms, but they do not automatically demonstrate engineering scalability. The transition from microscale or nanoscale specimens to macroscopic components introduces new problems: maintaining dimensional precision over large volumes, avoiding accumulated defects, preserving node integrity, controlling residual stresses, and ensuring reproducibility [35,40,47]. In addition, nanolattices are often evaluated primarily under compression, whereas structural applications may involve tension, shear, bending, cyclic loading, impact, environmental degradation, or complex multiaxial stress states [22,35,38]. Thus, nanolattices should be viewed less as immediately deployable structural materials and more as controlled demonstrations of how architecture and size effects can be combined to alter the density–strength relationship [22,35].

Ceramic nanolattices provide one of the clearest examples of architecture modifying the apparent mechanical behavior of a brittle material. Ceramics are intrinsically stiff and strong but are typically limited by brittle fracture and flaw sensitivity. Hollow ceramic lattices challenge this limitation by changing the sequence of deformation events. When wall thickness, radius, and topology are properly selected, deformation can proceed through shell buckling or wrinkling before catastrophic fracture occurs. This is a crucial insight [21]. Brittleness is not only a constituent-material property; it is also a structural failure-mode problem. If an architecture concentrates tensile stresses at nodes or walls, brittle fracture will dominate [21,35,48]. If it instead promotes stable local buckling before fracture, even a brittle ceramic can display reversible and energy-absorbing deformation [21].

However, this architectural transformation should not be overstated [21,35]. Ceramic metamaterials do not make ceramics intrinsically ductile. Rather, they create a narrow mechanical window in which instability precedes fracture [21]. Outside this window, small changes in wall thickness, defect population, node geometry, or loading condition may return the structure to catastrophic failure. This makes the design of recoverable ceramic metamaterials highly conditional. Their promise lies not in eliminating brittleness, but in controlling when and where fracture occurs relative to buckling [21,35]. For this reason, future work should focus not only on maximum recoverable strain or compressive strength, but also on statistical reliability, cyclic durability, imperfection sensitivity, and scalability of the failure-mode control mechanism [21,35,40,48].

Inverse opal architectures occupy an intermediate position between discrete lattices and shell-based metamaterials. Their curved, interconnected surfaces provide quasi-continuous load-bearing pathways that differ from both random foams and strut networks. Mechanically, this is attractive because curved surface networks can distribute stresses more uniformly and engage more material in load transfer than isolated struts. In this respect, inverse opals reinforce a broader conclusion: efficient mechanical performance does not require straight members or conventional truss logic [43]. What matters is whether the architecture promotes favorable stress distribution and suppresses inefficient localized deformation [33,43]. Nevertheless, inverse opals also highlight a recurring challenge in the field. Mechanical superiority under one loading mode does not imply universal superiority [35,43]. A structure optimized for compression may not perform equally well under tension, shear, fatigue, or impact [35,38,43]. Moreover, coating quality, wall continuity, anisotropy, and local curvature can strongly influence failure [35,43]. The value of inverse opals therefore lies not in replacing other architectures, but in demonstrating that surface-mediated load transfer is a viable alternative to strut-dominated design [43].

Recent shell-based architectures further support the idea that surface-dominated stress transfer may offer a more efficient route to lightweight performance than increasingly complex truss networks. By introducing curved surfaces into open or semi-open frameworks, such designs attempt to combine the mechanical advantages of shell action with the practical benefits of accessible porosity [44]. This is especially important because fully closed-cell structures, although mechanically attractive, can be difficult to fabricate, inspect, functionalize, or use in applications requiring permeability or internal access [40,44]. Semi-open shell architectures therefore represent an important compromise between structural efficiency and manufacturability. Yet their success depends on more than replacing struts with surfaces [44]. Curvature, shell thickness, junction geometry, and imperfection tolerance must be designed so that membrane stresses are activated without triggering premature buckling or fracture [35,40,44]. The key challenge is not simply to maximize surface area, but to achieve stable membrane-dominated deformation over a useful strain range [35,44].

### 3.4. Hierarchical Organization, Fractal-Inspired Design, Porosity, and Dynamic Performance

Hierarchy introduces an additional level of complexity by distributing structural function across multiple length scales [17,20,49,50]. In single-scale metamaterials, the principal design variables are typically unit-cell geometry, relative density, and constituent material [16,34,36,50]. Hierarchical systems add further variables: how stiffness, porosity, connectivity, instability, and energy dissipation are allocated among scales. This changes the design problem from optimizing a repeating unit cell to programming a multi-scale mechanical response. The macroscopic properties of hierarchical metamaterials cannot be predicted from relative density alone because each level modifies the effective behavior inherited by the next. Connectivity, scale ratio, fractal dimension, local stiffness contrast, and damage pathways all become important. This explains why hierarchical architectures may deviate from classical cellular-solid scaling laws and display mechanical behavior that appears anomalous when viewed through a single-scale framework [17,20,49,50].

The biological motivation for hierarchy is compelling but should be interpreted critically [17,20,35]. Natural materials such as bone, nacre, enamel, shells, spider silk, and diatom structures achieve remarkable combinations of stiffness, strength, toughness, and damage tolerance through nested organization [17,20,48,51]. However, their performance does not arise from complexity alone. It arises because different structural levels perform distinct mechanical roles: some transfer load, others deflect cracks, dissipate energy, arrest damage, or accommodate deformation. Engineered hierarchical metamaterials should be judged by the same criterion. A hierarchy that simply repeats geometry without assigning mechanical function may add manufacturing difficulty without improving performance. Worse, it may introduce weak junctions, stress concentrations, excessive compliance, or defect-sensitive features. Thus, hierarchy should be treated as a design strategy, not as an inherent advantage [17,20,35,52].

This point is particularly important for fractal-inspired metamaterials. Fractal geometry is often attractive because it offers self-similar organization across scales, but self-similarity does not automatically imply superior mechanical performance. The effect of fractality depends on the intended function and the deformation mode it promotes [47,50]. In stretchable electronics, for instance, fractal-inspired interconnects can be useful precisely because they reduce tensile stiffness and delocalize strain. This is beneficial when compliance, conformability, or mechanical comfort is required [47]. In load-bearing structural materials, however, the same tendency toward bending compliance may be undesirable if the goal is high stiffness or strength. Therefore, fractal design should not be presented as a universal route to stronger or stiffer materials [47,50]. It is more accurately understood as a method for programming mechanical response, whether that response involves compliance, strain redistribution, energy absorption, wave attenuation, or damage tolerance [38,47,50]. A related but mechanistically distinct family comprises spinodal metamaterials, whose bicontinuous, tunable-length-scale morphology is generated by solving the nonlinear Cahn–Hilliard equation under periodic boundary conditions rather than by recursive geometric construction [29]. Spinodal architectures should not be classified as fractals unless a genuine fractal dimension or scale-invariant behavior is demonstrated; nevertheless, they illustrate an alternative, partial-differential-equation-based route to multiscale topology generation and tailored homogenized elastic properties that complements the recursive hierarchical and fractal-inspired constructions that are the focus of this review.

Porosity further illustrates why scalar descriptors are insufficient for critical evaluation [17,43,50]. In many studies, porosity or relative density is treated as the primary structural variable [16,43,50]. Yet two materials with the same porosity can exhibit very different stiffness, strength, permeability, and failure behavior if their pore networks differ in connectivity, anisotropy, roughness, curvature, and size distribution. In hierarchical and fractal-inspired systems, void space is not merely removed material; it is an active architectural parameter [17,43,50]. Large connected pores may concentrate stress and accelerate failure, whereas smaller distributed pores may reduce weight while promoting crack deflection or progressive damage [17,35,43,50]. Self-similar pore networks may influence both mechanical and transport properties in ways that cannot be captured by total porosity alone. Therefore, meaningful characterization of hierarchical porous metamaterials should include not only relative density, but also pore topology, connectivity, scale distribution, and their influence on stress localization [17,40,43].

Dynamic mechanical performance expands the relevance of hierarchy even further [38]. Many metamaterials are evaluated using quasi-static compression, but real applications often involve vibration, impact, cyclic loading, shock, or elastic wave propagation [35,38]. Hierarchical architectures are attractive in these contexts because different length scales can interact with different wavelengths or frequency ranges. A larger-scale framework may provide global stiffness and structural integrity; intermediate features may control buckling, collapse pathways, or progressive damage; smaller-scale features may contribute to damping, scattering, or crack deflection [38]. Such multi-scale role assignment is difficult in single-scale lattices and represents a strong argument for hierarchical design [17,20,38].

However, dynamic functionality also introduces additional uncertainty [35,47]. Wave attenuation, band-gap formation, impact resistance, and damping depend strongly on boundary conditions, loading rate, viscoelasticity, imperfection wavelength, and manufacturing variability [35,38,40]. A metamaterial that behaves predictably under slow compression may deform very differently under high-rate loading. Under dynamic conditions, inertia and local stress states can suppress some instability modes while amplifying others. Unit-cell size, pore spacing, shell curvature, strut length, and hierarchy ratio may therefore act as wavelength-selection parameters, determining which deformation or failure modes dominate. For this reason, dynamic characteristics should be evaluated under conditions corresponding to actual applications, rather than being determined solely on the basis of quasi-static stiffness or strength [35,38].

Across the field, several unresolved challenges continue to limit the transition of mechanical metamaterials from laboratory demonstrations to reliable structural materials [35,36,38,40]. First, many exceptional property values are obtained from small, carefully fabricated specimens [22,35]. These results are valuable for identifying physical mechanisms, but they do not automatically establish scalability [22,35,40]. Second, comparisons across studies are often complicated by differences in specimen size, fabrication route, boundary conditions, strain rate, density definition, modulus extraction, and failure criterion [35,36]. Third, real failure is frequently governed by imperfections rather than ideal topology. Node fracture, wall-thickness variation, surface roughness, residual stress, misalignment, and local shell instability may dominate the measured response [21,42,47,50]. Fourth, there is a persistent trade-off among stiffness, strength, recoverability, energy absorption, damping, and manufacturability [35,38,40,44]. Architectures optimized for stiffness may fail abruptly, whereas architectures optimized for recoverability may rely on compliant mechanisms that reduce load-bearing capacity [21,35,44].

For these reasons, the decisive metric for future mechanical metamaterials should not be the stiffness-to-density ratio alone [35,38]. Specific stiffness and specific strength remain important, but they capture only part of the design problem [16,22,47]. A structurally useful metamaterial must sustain stable, progressive, and damage-tolerant deformation under realistic service conditions. It must tolerate imperfections, maintain performance over relevant length scales, and behave reliably under the loading modes for which it is intended. This requires a multi-objective design approach in which topology, geometry, material selection, feature size, manufacturing route, imperfection tolerance, and failure mode are optimized together [35,38,40].

Overall, the reviewed literature indicates that mechanical metamaterials are best understood as systems for controlling deformation mechanisms rather than as materials defined by unusual geometry alone [16,17,18,19,20,21,22,33,34,35,36,38,39,40,41,42,43,44,46,47,49,50,51,53]. Stretch-dominated lattices reduce the density penalty of conventional foams by promoting axial load transfer [16]. Nanolattices exploit size-dependent strengthening and flaw reduction [22]. Hollow ceramic architectures demonstrate that brittle fracture can, under suitable conditions, be preceded by reversible shell buckling [21]. Inverse opals and shell-based designs show the value of surface-mediated stress distribution [43,44]. Hierarchical and fractal-inspired architectures broaden the design space by assigning mechanical functions across multiple length scales [17,20,38,47,49,50]. Yet none of these strategies is universally optimal [33,35]. Each represents a different compromise among stiffness, strength, recoverability, damping, manufacturability, and imperfection sensitivity [33,34,35,40,44].

The most promising future direction is therefore not the pursuit of a single superior architecture, but the integration of complementary mechanisms. High-performance metamaterials will likely combine micro- or nanoscale defect control, stretching- or membrane-dominated load paths, optimized node and shell geometry, hierarchical energy dissipation, and manufacturable open or semi-open architectures [16,17,20,21,22,33,34,35,36,38,40,41,42,43,44,47,49,50]. For critical review purposes, the field should move beyond describing architectures in terms of visual complexity, periodicity, or relative density alone [17,33,35,50]. The essential questions are mechanistic: how does geometry redirect load paths, how does it modify deformation mode, where does failure initiate, how stable is the post-buckling or post-yield response, and how robust is the architecture to imperfections and scale-up [21,33,35,38,40,44]? Only by addressing these questions can mechanical metamaterials progress from impressive small-scale demonstrations toward reliable structural and multifunctional materials [35,36,38,40].

**Figure 4:**
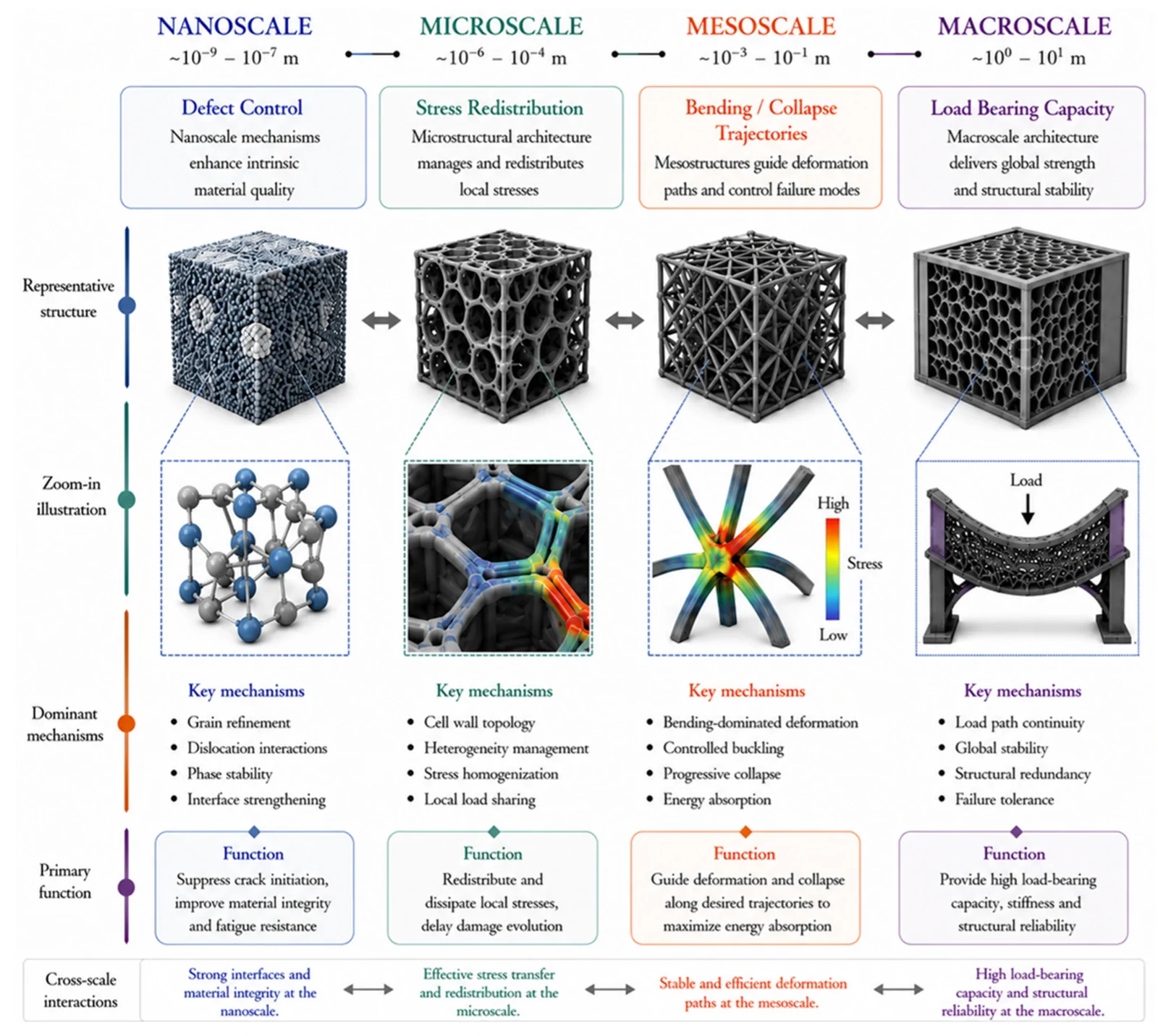
Multiscale structure–function relationships in hierarchical mechanical metamaterials. Conceptual framework synthesized from representative multiscale studies [53,54,55,56,57,58,59,60,61,62,63,64,65]. The figure was created with assistance from ChatGPT (OpenAI) GPT-5.6 Luna and subsequently reviewed, edited, and scientifically validated by the authors.

**Figure 5:**
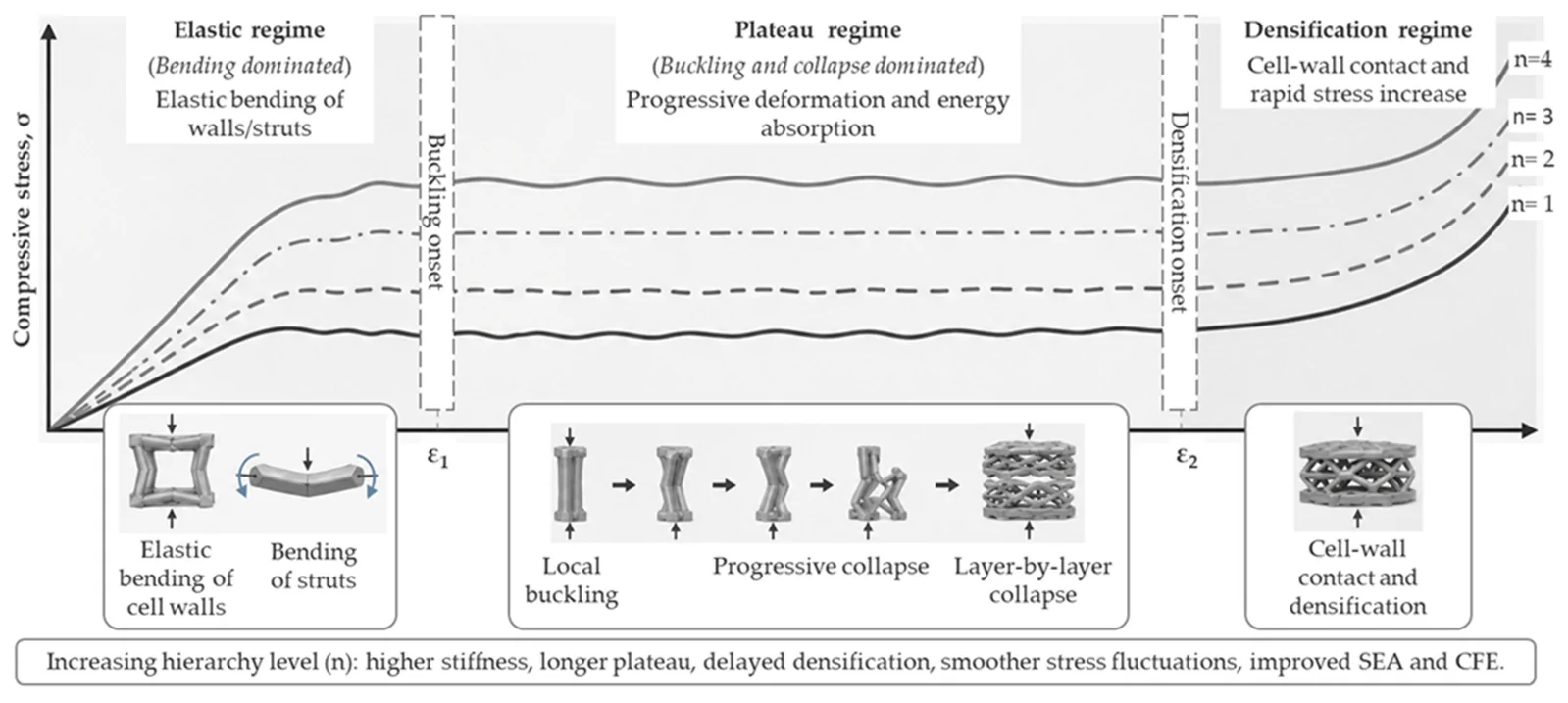
Schematic compressive stress–strain response and dominant deformation mechanisms of single-scale and hierarchical cellular metamaterials. Increasing hierarchy level, n, is associated with increased stiffness, an extended and more stable plateau response, delayed densification, smoother stress fluctuations, and enhanced specific energy absorption and crush force efficiency. The curves provide a qualitative, literature-informed conceptual synthesis of bending-to-buckling transitions reported for hierarchical cellular metamaterials and are not intended as quantitative data for a specific architecture [16,37,54,55,57,58,66]. The direction of trends is not universal and depends on material, loading mode, relative density, and manufacturing defects. The figure was created with assistance from ChatGPT (OpenAI) GPT-5.6 Luna and subsequently reviewed, edited, and scientifically validated by the authors.

The mechanistic relationships discussed above are schematically summarized in Figure 4 and Figure 5. Although the presented fractal-inspired architectures possess comparable low relative densities, their compressive responses evolve through different deformation pathways because of differences in multiscale topology. Consistent with the established mechanical behavior of cellular solids, the compressive response can be divided into three characteristic regimes: an initial elastic regime dominated by bending of cell walls or struts, a plateau regime associated with progressive local buckling and sequential layer-by-layer collapse that governs energy absorption, and a final densification regime characterized by cell-wall contact and a rapid increase in stress as the available pore volume is exhausted [37,54,55]. Increasing the hierarchical level delays the onset of densification, extends the plateau region, reduces stress oscillations during progressive collapse, and consequently improves both the specific energy absorption (SEA) and crushing force efficiency (CFE), indicating a more stable and efficient collapse process [33,35,40,56]. Collectively, these figures emphasize that the mechanical performance of fractal-inspired metamaterials is governed primarily by the interaction between hierarchical architecture, deformation mechanisms, and damage evolution, rather than by relative density alone.

## 4. From Nanoscale Defect Control to Macroscale Programmability: Functional Roles of Hierarchy and Instability

### 4.1. From Programmable Stiffness to Programmable Response

The mechanical interpretation of hierarchical, fractal-inspired, and instability-driven metamaterials is undergoing an important transition. Early architected materials were commonly assessed through density-normalized stiffness and strength, with the main design objective being the replacement of inefficient bending-dominated deformation by stretching- or membrane-dominated load transfer. Although this remains a useful baseline, recent literature shows that many important advances no longer arise from stiffness maximization alone. Instead, contemporary metamaterials increasingly use hierarchy, recursive or hierarchical topology, nanoscale architecture, buckling, plasticity, and symmetry control to prescribe how a structure carries load, redistributes stress, dissipates energy, undergoes instability, and recovers after large deformation [53,54,55,56,57,58,59,60,61,62,63,64,65]. In this sense, the mechanical performance of such systems should be evaluated less as a fixed point on a modulus-density or strength-density chart and more as a programmable structure-level response. A structure that exhibits moderate initial stiffness but stable progressive collapse, recoverability, and controlled energy dissipation may be more useful in many engineering contexts than one that maximizes elastic modulus but fails abruptly. This distinction is central to the critical evaluation of hierarchical and fractal-inspired metamaterials.

**Figure 6:**
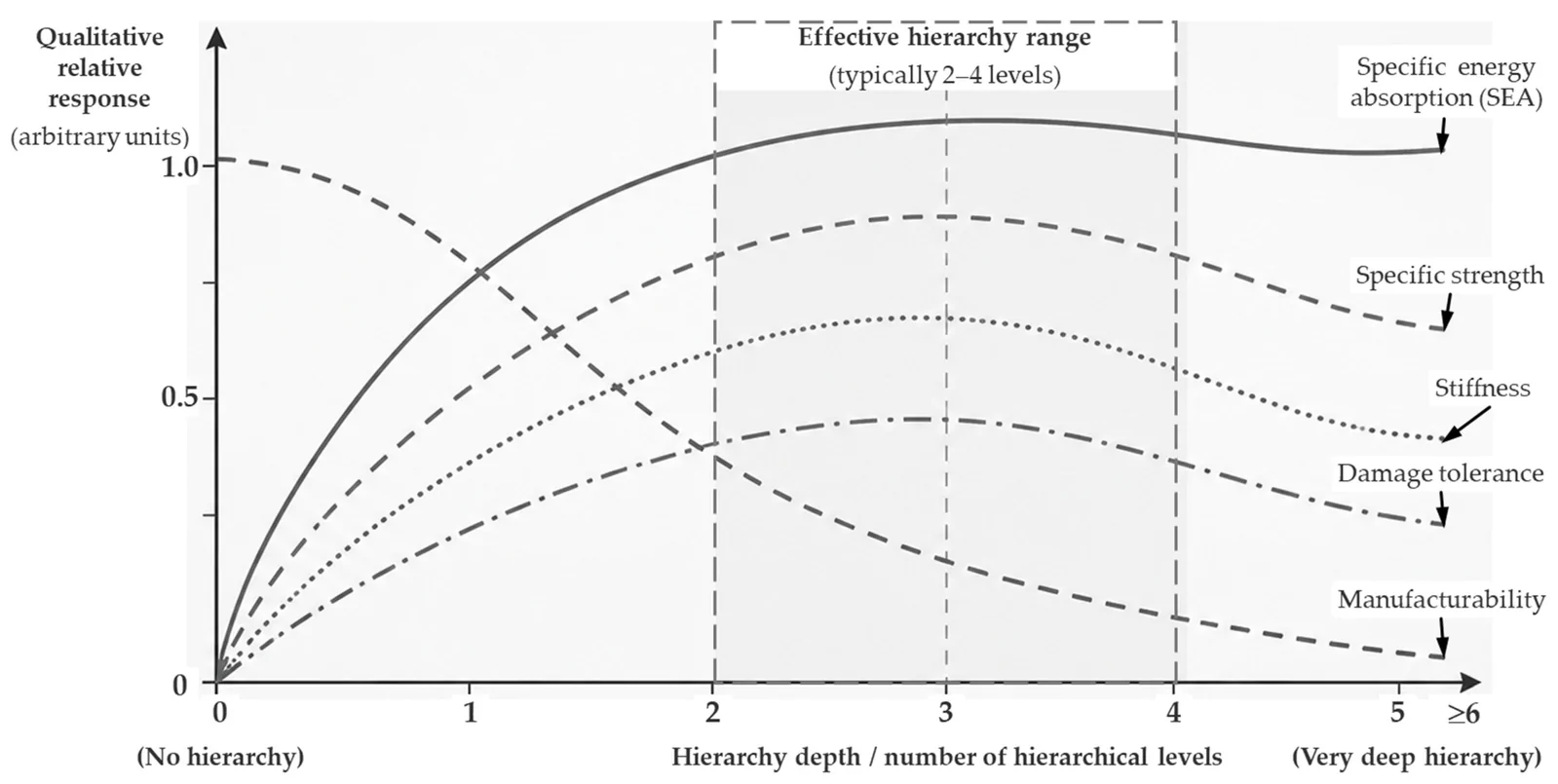
Qualitative trade-off between hierarchy depth and mechanical performance in hierarchical and fractal-inspired metamaterials. Intermediate hierarchy levels are schematically associated with improved energy absorption, stiffness, specific strength, and damage tolerance, whereas excessive hierarchy may compromise manufacturability. The trends are synthesized from representative multiscale studies [20,53,54,55,56,57,58,59,60,61,62,63,64,65,66] and should be interpreted as general structure–property tendencies rather than quantitative predictions for specific architectures.

A key conceptual advance is the recognition that mechanical hierarchy is beneficial only when each structural level performs an active mechanical function. Crystallography-inspired multiscale metamaterials illustrate this principle particularly well. By translating crystallography-derived topologies into architected systems, such designs connect crystallographic motifs, relative density, topology, and deformation mechanisms across scales. The significance of this approach is not the visual resemblance to atomic crystals, but the possibility of using crystallographic concepts—orientation, topology, grain-like domains, and boundary-controlled deformation—as design variables in architected solids. At the nanoscale, molecular dynamics simulations of nickel-based nano-architected metamaterials show that free surfaces can promote dislocation nucleation while restricting dislocation propagation, leading to flow stresses exceeding those of bulk counterparts [53]. This dual role is mechanistically important: nanoscale architecture can reduce the strain required for plasticity while simultaneously preventing defect motion from developing into long-range slip. Therefore, the frequently invoked “smaller is stronger” argument should be treated critically. It is not only the absence of large flaws that matters, but also the topology-mediated confinement of plasticity and the redistribution of defect activity by free surfaces and internal boundaries. The resulting view of hierarchical mechanical metamaterials is therefore fundamentally functional rather than purely geometric. Hierarchical organization becomes mechanically advantageous only when different structural scales contribute distinct and complementary roles to the overall deformation process. In this framework, nanoscale features primarily influence defect activity and damage initiation, microscale architecture governs stress redistribution and load sharing, mesoscale topology controls deformation trajectories and instability pathways, whereas macroscale organization determines global load-bearing capacity and structural integrity. Mechanical performance therefore emerges from the coordinated interaction of mechanisms operating across multiple length scales rather than from any individual hierarchical level in isolation. This conceptual interpretation is illustrated schematically in Figure 6. The figure represents a conceptual framework intended to illustrate structure–function relationships across scales rather than specific experimental observations or quantitative datasets.

The same principle applies to fractal-inspired hierarchical nanolattices, where performance enhancement emerges from the coordination of deformation across length scales rather than from hierarchy as a purely geometric feature. Hierarchical nanolattices based on self-similar architectures demonstrate near-theoretical scaling of strength and stiffness with relative density, together with high recoverability in hollow ceramic systems. However, the most important lesson from these structures is not simply that hierarchy improves stiffness or strength; rather, hierarchy changes the location and sequence of failure. In hollow ceramic nanolattices, brittle alumina does not become intrinsically ductile. Instead, the architecture redirects deformation into local beam buckling, shell buckling, microcracking at nodes, and distributed compliance, allowing hollow alumina samples to recover up to approximately 98% of their original height after compression to strains of at least 50%. This is a clear example of architecture-enabled failure-mode control: global recoverability is achieved by allowing local deformation modes to activate in a controlled manner. Yet the same work also shows that additional hierarchy does not necessarily improve mechanical performance; hierarchy beyond the second order did not increase stiffness or strength [21,53,57]. This observation is fundamental for design: hierarchy has an optimum in this design context. Beyond that optimum, additional structural levels may increase node density, geometric imperfection sensitivity, and local compliance without improving load transfer. While hierarchy is frequently associated with improved mechanical performance, the relationship is not necessarily monotonic. Additional hierarchical levels can activate new deformation mechanisms, improve stress redistribution, enhance energy absorption, and increase damage tolerance. However, excessive hierarchy may introduce manufacturing complexity, geometric imperfections, and inefficient load transfer pathways that ultimately reduce the net performance benefit. Consequently, the mechanical advantages of hierarchy are expected to follow an optimum rather than an indefinitely increasing trend. This conceptual interpretation is illustrated schematically in Figure 6.

Macroscopic hierarchical mechanical metamaterials strengthen this conclusion by demonstrating that useful structural performance may require a deliberate combination of deformation modes. Hierarchical systems fabricated using stereolithography and combining stretch-dominated octet-truss architectures with bending-dominated cubic architectures show enhanced toughness and energy absorption, along with zigzag compressive stress–strain behavior. The critical implication is that bending-dominated mechanisms should not be dismissed as mechanically inferior in all contexts. For purely stiffness-limited design, bending typically reduces efficiency; however, for energy absorption, damage tolerance, and progressive deformation, bending can be advantageous when integrated into a broader architecture dominated by more efficient load paths. The reported UAV demonstrator, approximately 85% lighter than the bulk counterpart and associated with markedly extended flight duration, illustrates that hierarchical metamaterials are moving toward application-level design rather than remaining abstract mechanical specimens [57]. Nevertheless, such translation also exposes unresolved challenges. As hierarchy becomes more elaborate, node complexity, print resolution, surface roughness, and accumulated geometric defects increasingly affect performance. Thus, manufacturability is not a secondary issue but part of the mechanical design problem itself.

Fractal assembly provides a complementary pathway by shifting attention from top-down unit-cell repetition to bottom-up control of connectivity. Dual-anisotropic ferromagnetic polymer particles with independently tunable shape and magnetic response can form local interaction motifs, including head-to-tail, side-by-side, and orthogonal T-junction configurations, which evolve into scale-invariant fractal-like networks [56]. Although such colloidal assemblies are not primarily presented as structural load-bearing metamaterials, they are highly relevant to future mechanically functional fractal-inspired materials. They show that multiscale architecture can arise from programmed local junction rules rather than from imposed periodic geometry. This distinction matters because many natural hierarchical materials are not perfectly periodic; their performance often derives from controlled irregularity, distributed connectivity, and imperfection-tolerant networks. The challenge for future fractal-inspired metamaterials is therefore to translate assembly-level statistical connectivity into predictable stiffness, strength, and damage evolution. Without that link, fractal morphology may remain descriptive rather than mechanically predictive.

### 4.2. Instability, Plasticity, and Data-Driven Design

Instability-driven metamaterials further broaden the meaning of load-bearing capacity by treating buckling as a functional mechanism rather than a failure event. Buckling-based architectures can exhibit switchable stiffness, reversible energy dissipation, programmable deformation, and tunable dynamic response [60,61]. The critical point is that such structures operate in a mechanical regime where effective properties are path-dependent. Findeisen et al. showed that metamaterials built from unstable buckling elements can display programmable or switchable behavior and reversible energy dissipation, distinguishing them from conventional plastic and viscoelastic materials [58]. This has important implications for how strength and stiffness should be interpreted. In a conventional material, instability often marks the loss of load-bearing integrity. In an instability-driven metamaterial, local buckling can be the beginning of a useful transformation pathway. Therefore, peak load alone is insufficient: the stability of post-buckling branches, recoverability after unloading, and spatial distribution of activated cells become equally important.

The use of plasticity to tune buckling sequences adds another layer of design freedom but also introduces an important trade-off. Recent work demonstrates that yield area, yield criterion, and loading history can be used to tune buckling loads and sequential deformation patterns in metallic metamaterials. This is compelling because plasticity-enabled sequential metamaterials may combine low density, high stiffness, high strength, and high dissipation—properties that are difficult to combine in purely elastic systems [59]. However, the critical evaluation must distinguish between useful plasticity and irreversible degradation. Plasticity can stabilize sequential buckling and improve energy dissipation, but it may also reduce reusability, introduce residual deformation, and affect fatigue performance. Consequently, plasticity should not be viewed as a universal improvement over elastic buckling. It is more accurately understood as a design tool for applications where dissipation and programmed collapse are prioritized over full recoverability. For reusable vibration isolation or cyclic operation, elastic or elastomeric instability may remain more appropriate.

Dynamic metamaterials reveal another limitation of purely geometric design thinking. Buckling shells used as variable-stiffness resonators can generate tunable vibration bandgaps, but nominal symmetry does not guarantee mechanically symmetric deformation. Liu et al. showed that even symmetric buckling-shell configurations may deform asymmetrically under practical imperfections, causing sequential snap-through rather than simultaneous deformation. This finding is important well beyond the specific resonator design. It demonstrates that robust tunability depends not only on the target unit-cell geometry, but also on the stability of bifurcation pathways. The proposed superposition strategy for buckling shells provides a route to symmetrize deformation and achieve more reliable stiffness tuning and vibration bandgap control [61]. Similarly, metamaterials combining stiff square networks with periodically arranged voids show that critical buckling strain and buckling mode selection depend on the interaction between two periodic systems. Depending on the number and arrangement of voids in the unit cell, instability may preserve local periodicity or generate alternating patterns with new periodicity [62]. These results show that wave control, instability, and structural load bearing are not separate topics; they are coupled through geometry, symmetry, and bifurcation behavior.

The increasing complexity of these systems makes automated and data-driven design increasingly necessary. Hierarchical pentamode-like metamaterials designed using automated multiscale optimization demonstrate that hierarchical architectures can substantially improve bulk-to-shear modulus ratios compared with non-hierarchical or classical finite-joint pentamode alternatives [63]. Nevertheless, a high bulk-to-shear modulus ratio is not by itself a sufficient design goal for structural applications. If an architecture achieves an extreme ratio only by sacrificing absolute stiffness, strength, manufacturability, or robustness, its practical value may be limited. Thus, future optimization frameworks should target balanced property envelopes rather than isolated metrics. Symmetry-based datasets for mechanical metamaterials also indicate that machine learning may become useful for mapping symmetry–property relationships, especially in systems where buckling-induced pattern transformations break or modify initial microstructural symmetry [64]. However, data-driven design will be useful only if datasets incorporate physically meaningful outputs, including instability modes, post-buckling response, and imperfection sensitivity, rather than elastic constants alone. Beyond symmetry-based datasets, broader deep-learning frameworks—spanning forward property prediction, inverse design, and generative modeling—are increasingly used to navigate the architecture–property space of mechanical metamaterials [65].

Taken together, the reviewed literature indicates that the future of hierarchical, fractal, and instability-driven metamaterials lies in the assignment of distinct mechanical roles to different scales. Nanoscale features may confine defects and tune plasticity; microscale hierarchy may redistribute stress and delay localization; mesoscale buckling may control transformation pathways and energy dissipation; macroscale architecture may determine load-bearing capacity, recoverability, and dynamic response [23,53,54,55,56,57,58,59,60,61,62,63,64,65]. The central challenge is ensuring that added complexity remains mechanically purposeful. Hierarchy, fractality, and instability are not automatically beneficial. They become valuable only when they improve load transfer, stabilize deformation, enhance damage tolerance, or enable useful dissipation. Therefore, the next generation of mechanical metamaterials should be judged not by geometrical sophistication alone, but by the coherence of their structure–property–function relationships under realistic loading and manufacturing constraints.

## 5. Automotive Crashworthiness as a Case Study: Energy Absorption, Force Efficiency, and Multifunctionality

### 5.1. Crashworthiness Metrics and Design Trade-Offs

**Figure 7:**
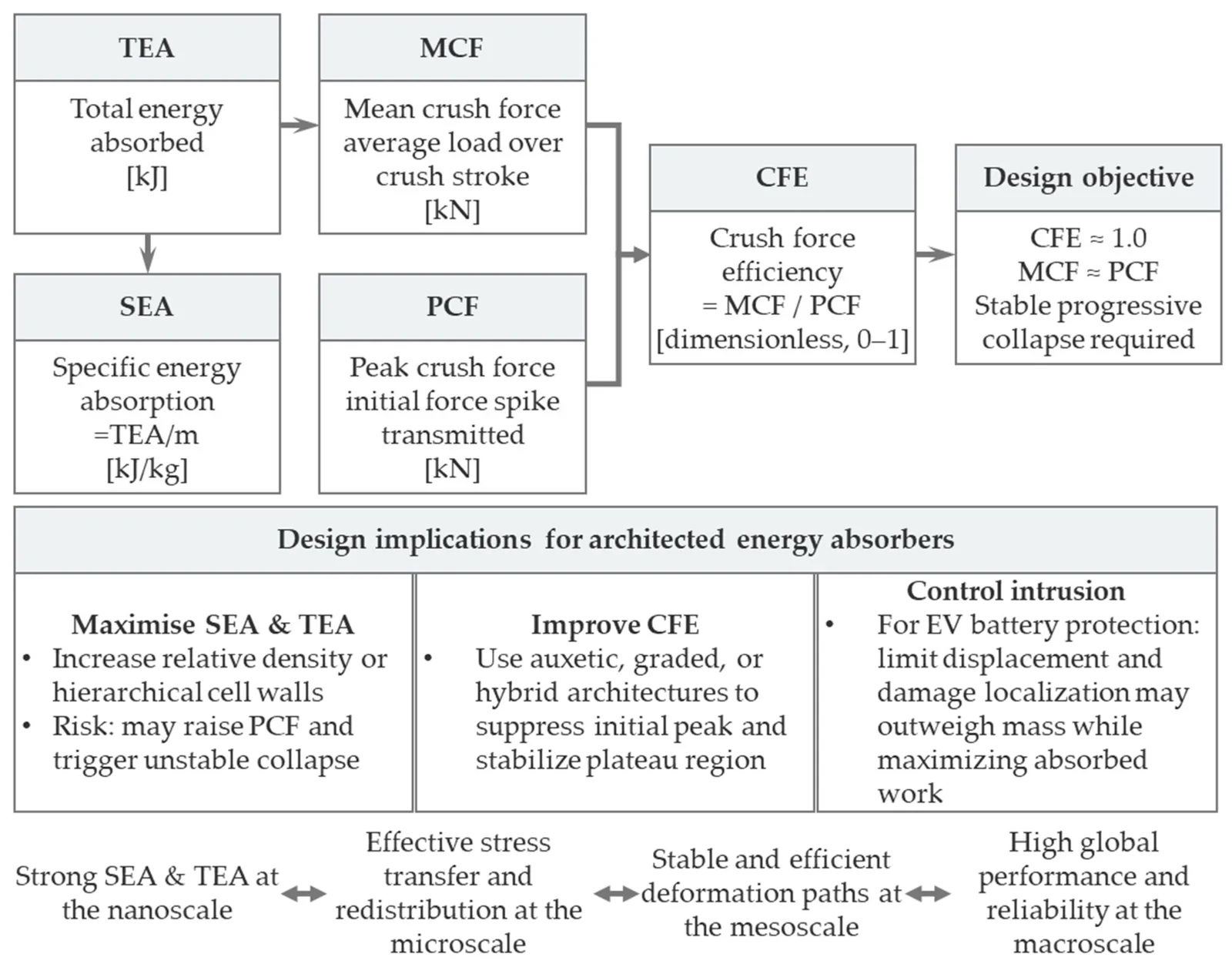
Conceptual framework illustrating the relationships among crashworthiness metrics (EA, MCF, SEA, PCF, and CFE) and their implications for the design of architected energy absorbers. The figure was created with assistance from ChatGPT (OpenAI) GPT-5.6 Luna and subsequently reviewed, edited, and scientifically validated by the authors.

The use of metamaterials and architected cellular structures in automotive crash management has moved the field beyond the conventional trade-off between stiffness and mass. In conventional metallic crash members, increasing load-carrying capacity often raises mass and, critically, the initial peak force transmitted to the vehicle structure. By contrast, auxetic lattices, honeycombs, hierarchical honeycombs, and cubic lattice-filled members offer a route to tailor deformation mode, plateau stress, and densification behavior through geometry rather than material substitution alone. This geometric controllability is the fundamental reason these structures are being explored for crash boxes, bumper systems, underfloor battery enclosures, side-impact battery protectors, and multifunctional battery casings that must also suppress vibration and shock. The most important implication for automotive design is that these architected absorbers are not simply “lightweight crush fillers”; rather, they create a way to redistribute the balance among total energy absorption (TEA), mean crush force (MCF), peak crush force (PCF), specific energy absorption (SEA), and crush force efficiency (CFE), often with higher design freedom than monolithic thin-walled members [23,24]. The interdependencies among these metrics are illustrated in Figure 7. TEA is normalized by absorber mass to yield SEA; MCF and PCF together define CFE, which measures how uniformly load is sustained over the crush stroke. A structurally useful absorber must simultaneously maximize SEA, maintain high MCF, suppress PCF, and achieve CFE approaching unity—objectives that often conflict and require architecture-specific trade-off management.

Automotive crashworthiness is used as a case study in this review, rather than aerospace energy absorption, flexible robotics, or generic impact protection, for three reasons specific to its load characteristics and structural failure modes. First, automotive crash members operate under a narrow, standardized and well-characterized loading regime (axial and oblique quasi-static to moderate-rate crushing, typically 1–15 m/s), for which the largest and most directly comparable body of architected-metamaterial literature exists [23,24,25,26,67,68,69,70,71,72,73,74,75,76,77,78,79,80,81,82]. It allows a more systematic mechanics comparison than is currently possible for aerospace or robotic applications, where reported loading conditions and metrics are far less standardized. Second, automotive structures combine, in a single component, the requirement for energy absorption, mass efficiency, and low-cost, high-volume manufacturability, so that the trade-offs between hierarchy-enabled performance and manufacturing sensitivity discussed throughout this review are especially visible and consequential in this application. Third, the emergence of underfloor battery enclosures in electric vehicles has created a multifunctional design problem—simultaneous crash protection, vibration attenuation, and thermal management. That illustrates the structure-mechanism-property-function argument developed in this review more concretely than a single-objective case study would. Aviation energy absorption, flexible robotics, and general impact protection remain highly relevant applications and are noted throughout as directions for future work. However, they are not treated as the primary case study here because the mechanics-focused literature base for architected metamaterials in those domains is comparatively fragmented at present.

**Figure 8:**
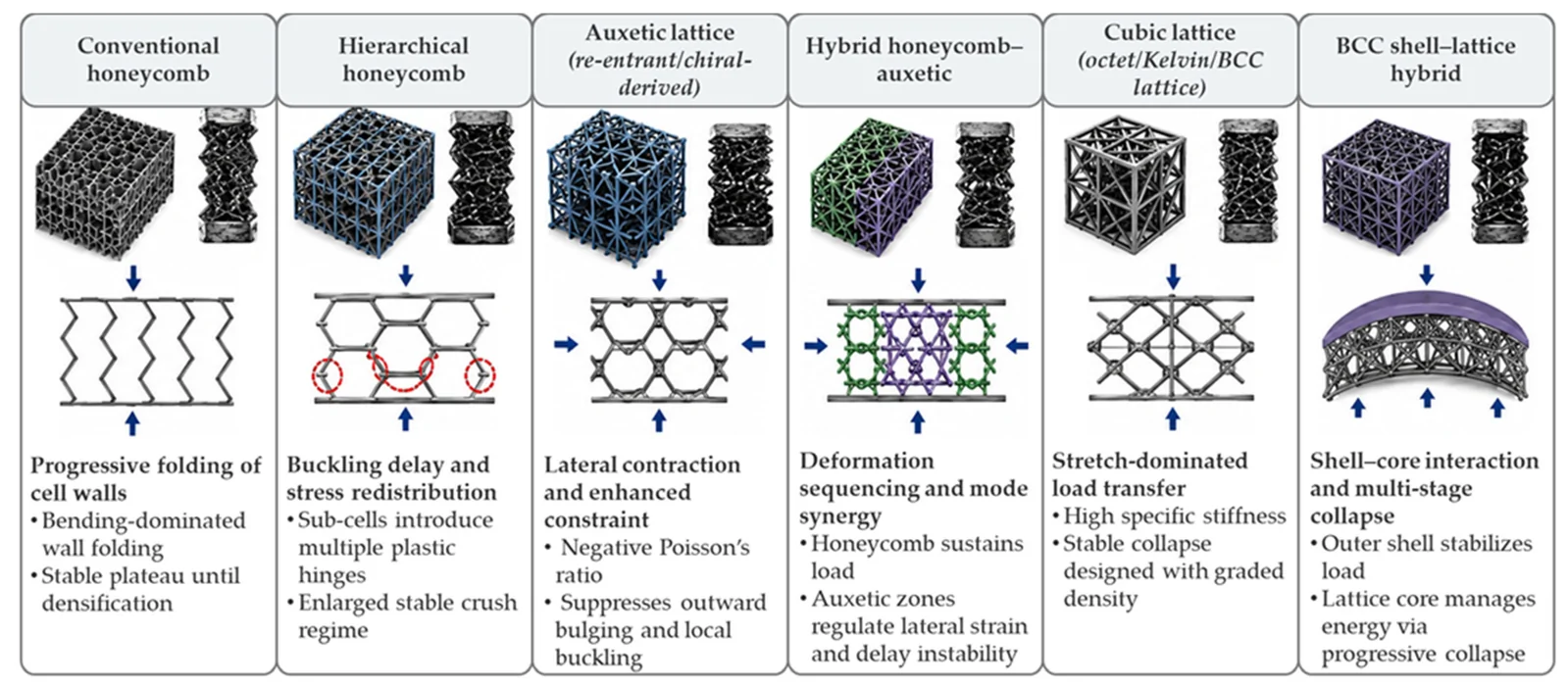
Architecture–mechanism relationships in cellular metamaterials. Representative cellular metamaterial architecture families and their dominant deformation, load-transfer, and collapse mechanisms, synthesized from studies [23,24,25,26,67,68,69,70,71,72,73,74,75,76,77,78,79,80,81,82]. This qualitative synthesis does not represent a single dataset and is not intended for quantitative extrapolation to a specific structure. The figure was created with assistance from ChatGPT (OpenAI) and subsequently reviewed, edited, and scientifically validated by the authors.

From a crashworthiness perspective, however, the literature consistently demonstrates that no single metric can fully characterize crashworthiness performance. TEA quantifies the total energy absorbed during crushing; SEA normalizes energy absorption with respect to structural mass; MCF represents the average load sustained during the crushing stroke; PCF governs the initial load spike and influences load transfer to adjoining vehicle structures, while CFE (=MCF/PCF) quantifies the uniformity of the crushing response by relating the sustained mean force to the initial peak force [23,24]. This distinction is crucial because many architected structures improve TEA or SEA primarily through increasing relative density or local stiffness; however, such gains can become counterproductive if they also increase PCF or promote unstable local collapse. Accordingly, the most promising automotive metamaterial concepts are not those exhibiting the highest nominal energy-absorption capacity in laboratory-scale tests, but those capable of sustaining progressive, stroke-efficient collapse while limiting load spikes and preserving intrusion constraints at the subsystem level [23,24,25]. The reviewed literature collectively indicates that automotive crashworthiness should be considered a multi-objective design problem rather than the maximization of a single performance metric. Different cellular and metamaterial architectures achieve improvements through distinct deformation mechanisms and therefore affect crashworthiness indicators in different ways. This architecture–mechanism–performance relationship is summarized schematically in Figure 8 and Table 1.

### 5.2. Honeycomb and Auxetic Architectures

A first robust pattern in the literature concerns honeycomb and hierarchical honeycomb fillers in crash members. Conventional honeycombs remain attractive because they offer relatively high stiffness-to-weight ratio, predictable plateau loading, and mature manufacturability. Yet hierarchy changes the problem qualitatively rather than incrementally: by introducing smaller sub-cells into the parent cell walls, hierarchical honeycombs delay catastrophic wall buckling, distribute plastic hinges more uniformly, and enlarge the stable crushing regime. This is why hierarchical architectures consistently outperform regular honeycombs in SEA and collapse control under out-of-plane loading. In optimization studies, the best hierarchical hexagonal configurations reached SEA values up to 148% higher than regular honeycombs, demonstrating that geometry alone can substantially alter crush efficiency [26]. For crash boxes and bumpers, the practical value of hierarchy is therefore not limited to “greater energy absorption”; it lies in the ability to maintain a longer and more stable plateau, which is directly relevant for improving MCF and CFE. The limitation, however, is equally clear: hierarchy increases geometric complexity, sensitivity to manufacturing deviation, and often the risk of rate-dependent behavior, so performance improvements reported in idealized numerical models may not transfer directly to stamped or mass-produced automotive components [24,26].

The second major trend concerns auxetic structures, especially re-entrant and chiral-derived lattices. Their negative Poisson’s ratio means that under axial compression they contract laterally rather than bulge outward, which can suppress premature sidewall buckling, enhance local constraint, and promote more stable folding in thin-walled enclosures [23,65]. This mechanism is attractive for crash boxes because it can improve MCF and CFE without necessarily requiring thick metallic walls. Yet the available evidence also shows that auxetic behavior alone does not guarantee superior crashworthiness. In equal-mass crash-box experiments, Simpson and Kazancı found that all cellular fillers improved the crush response relative to the empty tube, but the non-auxetic honeycomb filler produced the largest increase in energy absorption (79.1%), exceeding the gains from the auxetic-strut and re-entrant fillers (62.6% and 64.0%, respectively) [67]. Likewise, in square AA6063 crash boxes filled with PolyJet-printed lattices, anti-tetrachiral fillers outperformed re-entrant fillers in both SEA (+28.5% vs. +20.6% over the empty tube) and CFE (+50.6% vs. +40.27%) [68]. The critical conclusion is that the crashworthiness advantage of auxetic fillers is topology-specific and deformation-mode-specific: what matters is not the negative Poisson’s ratio as an isolated descriptor, but how that topology interacts with shell folding, contact evolution, and local instability. In other words, auxetic behavior is a useful design mechanism, not a universal ranking criterion [67,68].

This helps explain why hybrid concepts combining honeycomb and auxetic motifs are attracting increasing attention. A hybrid architecture can assign different structural roles to different cell families: positive Poisson honeycomb regions can sustain load efficiently and contribute to TEA, while auxetic regions can regulate lateral contraction, delay local buckling, and improve crushing regularity. The strongest studies are those showing that hybridization improves not only absorbed energy but also the quality of the crushing response. In a novel auxetic hierarchical crash box, Tan et al. demonstrated that combining a thin-walled outer tube with auxetic hierarchical filling cores improved crushing performance beyond both the traditional empty crash box and an aluminum-foam-filled alternative, and subsequent surrogate-based optimization further improved the crash response [25]. More recently, Yudhanto et al. reported that hybrid honeycomb–auxetic fillers in metallic crash boxes had minimal influence on the initial peak force but significantly improved progressive folding stability and mean crushing force, which is an especially attractive combination for automotive crash absorbers. Importantly, that study also found that filler material properties could influence the crushing response more strongly than topology itself [69]. This is a useful corrective to an overly geometry-centered literature: topology matters, but only within a broader system comprising base material, shell interaction, relative density, and manufacturing route [25,69].

### 5.3. Lattice-Filled Members and Multifunctional Battery Protection

A parallel development is the use of 3D lattice-filled thin-walled tubes, including cubic and body-centered cubic (BCC) lattices, to create shell–lattice hybrids. These structures are especially relevant to automotive crash rails and crash boxes because they exploit the synergy between external shell folding and internal lattice compression. Tao et al. showed that lattice-reinforced square tubes can exhibit clear interaction effects rather than a simple additive sum of shell and filler contributions: in their Materials study, the hybrid structure’s energy absorption increased by 43.40% relative to the sum of its individual components, while the best SEA rose by 83.02% relative to the empty tube [70]. In a related additively manufactured BCC-filled thin-walled tube, one-piece fabrication was not just a manufacturing detail but a crash mechanism enabler: under axial compression the SEA increased by 12.14% relative to split printing, and relative to the empty tube the designed hybrid delivered 112.60% higher SEA in axial loading and 580.15% higher SEA in transverse loading [71]. These results underline a critical, important point: in lattice-filled absorbers, the shell–filler interface and manufacturing integrity can be as important as unit-cell topology. For automotive use, this means that cubic or BCC lattices are promising not merely because they are lightweight, but because they can stabilize shell folding and raise MCF—provided debonding, print defects, and tolerance accumulation are controlled [70,71].

These architectures become particularly relevant in electric vehicle battery protection, where the design objective is not only to absorb impact energy but also to limit local intrusion, prevent cell rupture, and maintain pack integrity. In this context, high TEA is not necessarily the only dominant metric; controlling displacement and damage localization can be more important than maximizing absorbed work. This shift is evident in EV battery case studies using auxetic or hybrid cellular side members. Carakapurwa and Santosa optimized auxetic cellular protectors for pouch-battery systems and reported a 1220% increase in SEA over the baseline design, with the optimum obtained for a star-shaped auxetic geometry in aluminum [72]. Scurtu et al. showed that integrating a re-entrant auxetic energy absorber around a battery case could reduce the number of damaged cells by up to 35.2% at lower mass than the reference design [73]. At a larger subsystem level, Arslan and Karamangil designed a GFRP pultruded battery-box side profile with a hybrid honeycomb–auxetic geometry and reported crash performance improvements of approximately 360% over honeycomb-only structures and 88% over auxetic-only structures, while simultaneously reducing weight and cost in the optimized solution [74]. The broader lesson is that for EV battery protection, metamaterial design should be judged by a multi-objective intrusion–mass–load path framework rather than by SEA alone. A cellular concept with slightly lower SEA may still be superior if it produces a lower PCF, a more favorable damage path, or a stricter intrusion cap at the cell/module level [72,73,74,75].

A distinct but increasingly important branch of research concerns battery pack casings that must resist vibration and mechanical shock in service, in addition to crash loads. Although vibration isolation is not a crashworthiness metric in the narrow sense, it is highly relevant in EV structural integration because the same casing may be expected to provide both impact resistance and fatigue-sensitive protection against road-induced excitation. Mechanical metamaterial wall modifications have shown substantial vibration-mitigation potential. Lee and Lee reported that lattice and auxetic modifications could reduce vibration levels in aluminum battery pack casings by roughly 97–99%, although the gains were smaller in PPA-CF casings, indicating strong material–structure coupling [76]. In a subsequent study, Fan et al. showed that a novel hybrid auxetic battery casing outperformed a star-triangular honeycomb wall architecture, achieving simulated vibration reductions of 98.07% in the longitudinal, 95.09% in the vertical, and 93.60% in the transverse direction; experimental random-vibration attenuation was also substantial when damping add-ons were combined with the metamaterial casing [76]. The critical review point here is that multifunctionality introduces competing requirements: structural changes that increase local stiffness and shift natural frequencies upward may aid vibration suppression, but the same changes can increase PCF during crash loading. Therefore, future automotive battery enclosures should not treat crashworthiness and vibration isolation as separate optimization problems; they need coupled structural concepts that manage both impact energy and service-induced dynamic loads [76,77].

Vibration attenuation requires explicit treatment here as an application in its own right, rather than only as a future-work aspiration, because hierarchical and fractal-inspired honeycomb architectures already provide a demonstrated route to engineered band gaps and broadband wave attenuation at automotive-relevant length scales. Introducing a second, smaller-scale honeycomb into the walls of a parent honeycomb has been shown to open and widen low-frequency band gaps relative to the single-scale structure, with the sub-cell geometry acting as a locally resonant or wave-scattering feature [27,32]. Dual-functional hierarchical designs that combine a re-entrant or auxetic parent lattice with embedded resonant sub-features have demonstrated simultaneous vibration isolation and energy absorption from a single architecture, which is directly relevant to battery-casing and underbody applications where both functions are required from the same structural mass budget [30,31]. The mechanistic link to the rest of this review is direct: the same hierarchical load-path subdivision that improves crashworthiness also introduces the impedance mismatches and local resonances that create band gaps, so vibration attenuation and crashworthiness should be regarded as coupled, not independent, design objectives for hierarchical automotive structures.

### 5.4. Cross-Cutting Design Levers and Evidence Quality

Across these application areas, several design levers appear repeatedly. Relative density, wall thickness, and graded geometry remain the dominant controls on plateau stress, MCF, and SEA. Gradient design can be particularly effective because it allows deformation sequencing along the crushing path. For example, thickness-graded auxetic honeycombs can enhance energy absorption while also making PCF reduction more feasible in positive-gradient layouts, a combination that is especially valuable for automotive energy absorbers [78]. Likewise, gradient square honeycombs have shown improved SEA and dramatically improved CFE under selected loading regimes [79]. More recent auxetic topologies—notably combined-wall, vertex-based hierarchical, and arc-enhanced designs—confirm that local geometric refinement can simultaneously increase plateau stress, preserve the negative Poisson’s ratio effect, and improve SEA [80,81]. Yet this promising design space also exposes the field’s main practical bottleneck: most architectures exploit additive manufacturing to achieve fine geometric control, whereas automotive deployment still requires scalable, defect-tolerant, and low-cost manufacturing routes. In this sense, the transition from “mechanically elegant cell topology” to “automotive-credible component” remains incomplete [24,69,78,79,80,81,82].

The current knowledge base is therefore promising but methodologically heterogeneous (Table 2). Reviews of auxetic and bio-inspired structures repeatedly note a strong dependence on finite-element studies, limited experimental validation, and insufficient system-level testing under realistic combined loading states. Many studies still rely on quasi-static axial compression of coupons or simplified subcomponents, even though real automotive components experience oblique impacts, strain-rate effects, contact with adjoining structures, manufacturing imperfections, temperature variation, and, in EVs, possible thermo-electro-mechanical coupling [24,75]. Reported crash metrics are also not always directly comparable: some studies emphasize SEA, others TEA, others intrusion or damaged-cell count, and some omit PCF or CFE even though these are essential for judging whether a design is truly advantageous at the vehicle level [23,34,75]. Consequently, the literature does not yet justify a universally optimal topology. What it does justify is a more nuanced ranking: hierarchical honeycombs are strong candidates when maximizing SEA and collapse stability; auxetic fillers are attractive when lateral contraction and controlled collapse are needed; hybrid honeycomb–auxetic designs currently appear the most balanced option for improving MCF and CFE without a proportional PCF penalty; and cubic/BCC lattice-filled shells are particularly promising where shell–core synergy and additive manufacturing can be leveraged effectively [23,24,25,26,67,68,69,70,71].

Overall, the state of knowledge suggests that metamaterials and cellular architectures are most effective in automotive crashworthiness not when they are treated as stand-alone novel materials, but when they are integrated as load-path engineering tools. Their primary value lies in stabilizing deformation, extending the useful crush stroke, and raising MCF and CFE while keeping PCF and mass within acceptable bounds. The most convincing evidence presently supports hybrid and hierarchical concepts for crash boxes and battery-protection members, while lattice-filled metallic shells and multifunctional battery casings have opened a credible route toward future EV structural integration [25,69,70,71,72,73,74,76,77]. The next decisive step for the field is not the invention of increasingly elaborate unit cells, but the demonstration of manufacturable, rate-sensitive, subsystem-validated designs that can survive the transition from idealized metamaterial mechanics to automotive engineering reality [24,75].

## 6. Fractal Analytical Tools for Metamaterial Characterization: Fatigue, Surface Interaction, and Dynamic Response

### 6.1. Fatigue and Fracture: Crack-Path Complexity

**Figure 9:**
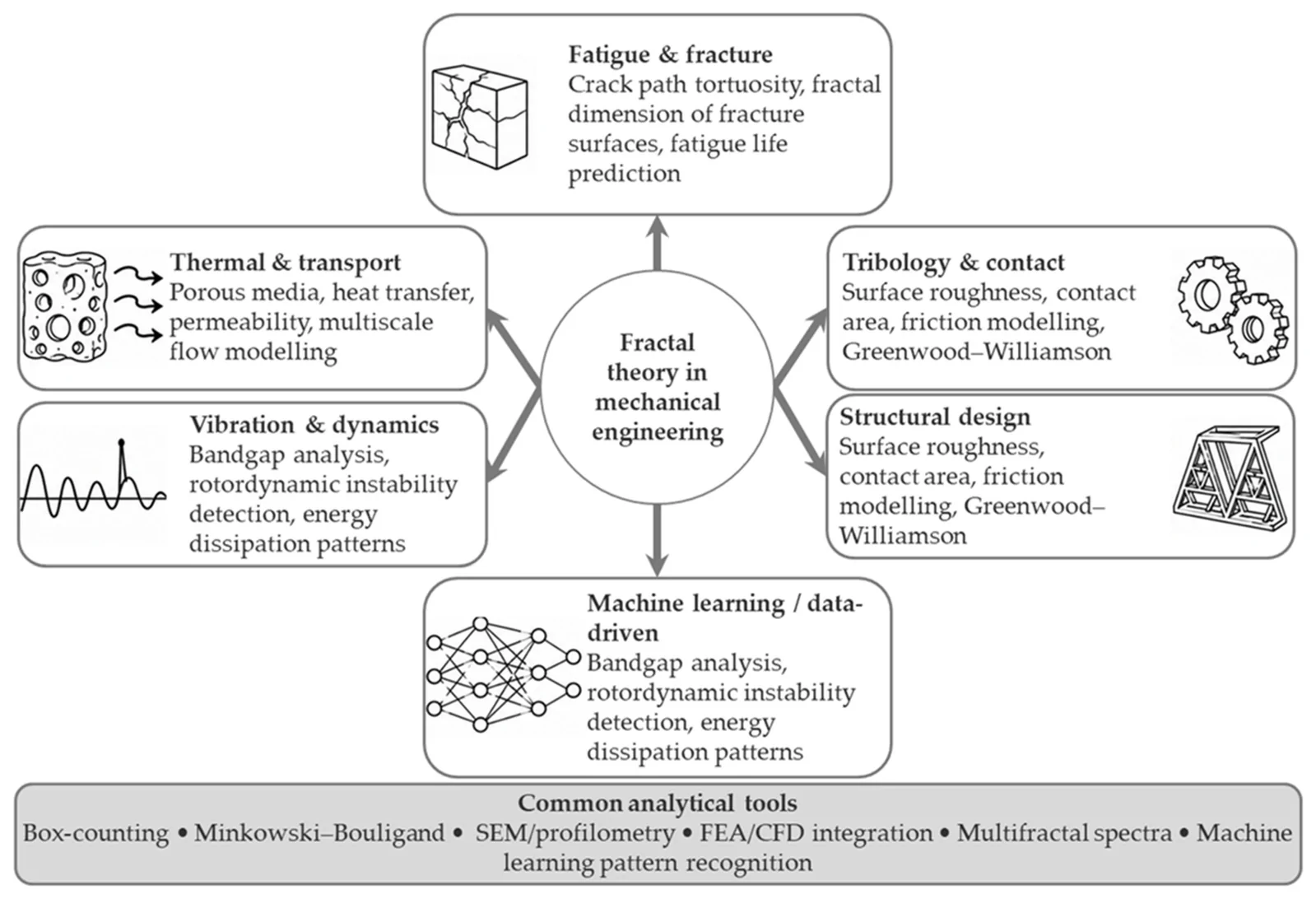
Conceptual map of fractal theory applications in mechanical engineering and the associated analytical tools. Domain groupings and analytical tools follow the classification in [84,85,86]. The figure was created with assistance from ChatGPT (OpenAI) GPT-5.6 Luna and subsequently reviewed, edited, and scientifically validated by the authors.

The integration of fractal theory into mechanical engineering has expanded the analytical tools available for describing complex material behavior, structural performance, and dynamic responses. Whereas traditional models often rely on Euclidean geometry and simplifying linear assumptions, fractal approaches provide a mathematically grounded way to describe irregular, multiscale phenomena that arise in fatigue, fracture, surface interaction, heat transfer, and vibration. The self-similar and scale-invariant characteristics of fractal structures can capture geometrical complexity and, where a validated relationship exists, link that complexity to functional performance across multiple physical domains [84]. As illustrated in Figure 9, fractal theory currently addresses five principal mechanical-engineering domains: fatigue and fracture analysis, tribology and contact mechanics, vibration and dynamics, structural design, and thermal transport. Common analytical tools, including box-counting algorithms, Minkowski–Bouligand methods, and integrated FEA/CFD workflows, support applications across these domains.

A particularly clear example of the value of fractal theory arises in fatigue and fracture mechanics. Fatigue cracks rarely propagate along smooth or predictable paths; instead, they develop tortuous, branching surfaces whose morphology challenges classical assumptions of regularity. Linear elastic fracture mechanics remains useful for macroscopic approximations, but it cannot fully describe the roughness and tortuosity evident at micro- and mesoscales. Fractal analysis addresses these limitations by quantifying crack-path complexity through fractal dimensions, which reflect surface irregularity and associated energy-dissipation mechanisms. Higher fractal dimensions may correspond to more intricate crack paths and greater dissipation during propagation, thereby slowing crack growth and extending fatigue life when the relationship is experimentally validated. This approach has supported refinements of crack-growth laws, improved life-prediction models, and a more detailed understanding of heterogeneous materials in which grain boundaries, inclusions, and voids influence crack trajectories [84,85].

Fractal-based approaches further enhance the capacity to identify critical crack lengths and growth transitions, leveraging scale-invariant features of fracture surfaces to relate microscale damage initiation to macroscale structural failure. The emergence of multifractal descriptions has provided additional nuance, capturing spatially varying complexity on fracture surfaces that single-dimension metrics cannot adequately represent. This evolution in modeling has direct implications for the design of fatigue-resistant systems, enabling engineers to anticipate failure more accurately and optimize material selection accordingly [84].

Beyond fracture analysis, fractal geometry has become useful in mechanical design, particularly where efficient material distribution and load management are required. Self-similar or recursively organized geometries can create lightweight structures by replicating nested patterns that redistribute stresses across scales. Fractal-inspired truss networks, branching structural elements, and hierarchical lattices may improve stiffness-to-weight ratios compared with conventional designs, which is relevant in aerospace, automotive engineering, and civil construction. In such architectures, recursive subdivision of load paths can mitigate stress concentrations, delay local failure, and place material where it is structurally needed. Biomimetic designs inspired by natural branching or vascular networks further illustrate how hierarchical organization can help manage mechanical demands [66,87,88,89].

### 6.2. Fractal Descriptors: Definitions and Mechanical Relevance

For the fractal descriptors used throughout this review to carry mechanical meaning rather than remain a qualitative label, they must be defined explicitly. The Hausdorff dimension D-H generalizes the notion of dimension to non-integer values and, for self-similar constructions, coincides with the similarity dimension

*D* = ln(*N*)/ln(1/*r*), (1)

where *N* is the number of self-similar copies generated at each iteration and *r* is the linear scale ratio between successive iterations [1,2]. In practice, *D* is most often estimated by the box-counting method, which covers the structure with a grid of boxes of size *ε* and counts the minimum number of boxes *N*(*ε*) required to cover it; *D* is then obtained as the slope of log *N*(*ε*) versus log(1/*ε*) over the scaling range in which this relationship is linear [84,85]. Two further descriptors are needed to make such an estimate physically meaningful for a finite engineering structure. First, the iteration order (or hierarchy depth) *n* and the scale ratio *r* together determine how many self-similar levels are actually realized before the recursive construction is truncated; unlike a mathematical fractal, an engineered hierarchical lattice has a finite-size cutoff—a smallest feature size set by manufacturing resolution—below which self-similarity cannot be extended, so the fractal dimension is only meaningful over the finite range of scales actually present in the manufactured part [86,90,91,92]. Second, lacunarity quantifies how non-uniformly the mass (or void space) is distributed at a given scale for structures that may share the same fractal dimension but differ markedly in appearance and, consequently, in stress distribution; it is typically estimated from the variance-to-mean-squared ratio of box masses across a gliding box of size *ε* [84]. Mechanically, a higher fractal or box-counting dimension in a fracture surface corresponds to a more tortuous crack path and correspondingly greater energy dissipation during propagation (Section 6.1), while in a load-bearing lattice, the iteration order and scale ratio jointly govern how many distinct length scales are available to redistribute stress and interact with a given loading wavelength (Section 3.4 and Section 5.3). This is the basis on which fractal descriptors are related, where applicable, to strength, stiffness, and dynamic response elsewhere in this review; where such a quantitative link has not yet been established in the literature for a given architecture, this review states so explicitly rather than implying one.

### 6.3. Tribology, Vibration, and Rotordynamics

Dynamic systems represent another domain in which fractal theory can provide useful insight. Vibrating mechanical elements, rotating machinery, and systems exhibiting nonlinear or chaotic responses frequently produce multiscale, irregular behaviors that are difficult to model using linear assumptions alone. Fractal dimensions can characterize the complexity of vibration patterns and reveal relationships in quasi-periodic or chaotic regimes. This capability may improve prediction of resonant behavior, energy-dissipation patterns, and the onset of instability. In rotating systems, fractal metrics have been used to identify early indicators of rotordynamic instability, offering a pathway toward more reliable machinery design [27,93].

Energy dissipation in dynamic systems often occurs heterogeneously and across multiple scales, making fractal-based descriptions useful complements to traditional linear damping formulations. These models can capture nonlinear interactions among vibration modes, microstructural mechanisms, and the influence of surface or interface complexity. As a result, they may support more precise predictions of failure modes, optimization of dynamic performance, and tailoring of system responses to demanding operating conditions [84].

### 6.4. Methodological Pillars: Numerical and Experimental Practice

The practical application of fractal theory relies on several methodological pillars that integrate advanced imaging, computational tools and multiscale modeling strategies. High-resolution surface characterization methods, such as SEM or profilometry, provide the data needed to compute fractal metrics through techniques like box-counting or Minkowski–Bouligand algorithms. These quantitative descriptors are then incorporated into established engineering models—such as Paris’ law for fatigue, Greenwood–Williamson contact models in tribology or CFD-FEA workflows for heat transfer and structural evaluation—thereby enhancing predictive fidelity without discarding existing analytical frameworks. Additive manufacturing offers an additional avenue for the realization of fractal structures, as modern fabrication technologies can reproduce hierarchical designs with high geometric precision, enabling practical implementation of fractal lattices and multiscale structural topologies [86,90,91,92]. Because this review synthesizes theoretical, numerical, and experimental studies, it is useful to be explicit about what rigorous practice looks like in each case, even though individual primary studies vary widely in how completely they report it. For numerical modeling, defensible practice includes explicit boundary conditions consistent with the intended loading mode, a mesh-sensitivity (convergence) study, clearly stated constitutive assumptions (elastic, elastic–plastic, or hyperelastic; rate dependence), explicit contact-modeling parameters at strut and shell junctions, and validation against at least one independent experimental or analytical benchmark. For experimental studies, defensible practice includes reporting specimen size relative to unit-cell size (to assess boundary and size effects), the manufacturing procedure and post-processing steps, an independently measured relative density (rather than the nominal design value), some form of defect characterization (e.g., CT scanning or optical inspection), a statement of repeatability (number of nominally identical specimens tested) and statistical dispersion, the loading mode and strain-rate range, and the associated measurement uncertainty. Not all studies cited in this review report every one of these items, and this is itself identified in Section 7 as a methodological gap limiting cross-study comparison.

### 6.5. Limitations and Outlook

Nevertheless, several challenges remain in bringing fractal theory into routine engineering use. Real-world systems rarely exhibit perfect fractality, so theoretical descriptors require careful interpretation and adaptation. Multifractal behavior, computational intensity, compatibility with conventional FEA/CFD tools, and limited experimental datasets present further hurdles. Addressing these issues will require hybrid computational methods, improved measurement techniques, and deeper integration between fractal mathematics and engineering practice [84].

Despite these challenges, current research suggests that fractal theory will continue to influence the design and analysis of mechanical systems. Machine learning and data-driven modeling provide new opportunities for identifying fractal patterns in complex datasets and optimizing hierarchical designs. Enhanced computational power and emerging fabrication technologies may also make multiscale fractal-inspired architectures increasingly feasible. In a broad sense, fractal approaches offer a useful conceptual and practical extension of metamaterial principles by emphasizing structure over composition and hierarchy over uniformity, thereby supporting the development of lighter, stronger, more efficient, and more resilient mechanical systems [87].

## 7. Challenges, Research Gaps, and Future Directions

### 7.1. Strength, Deformation, and Energy-Absorption Gaps

Despite the growing body of research on fractal and hierarchical metamaterials, several important challenges continue to limit their systematic development and practical implementation. A central issue is that many studies emphasize unusual or effective properties, while comparatively less attention is devoted to the mechanics that govern how such properties arise internally. In particular, the links between hierarchical geometry, stress redistribution, local instability, and failure evolution remain insufficiently understood. Addressing these limitations requires not only identifying open questions, but also outlining concrete directions along which the field can mature into a predictive, engineering-oriented discipline; both aspects are therefore considered jointly in this section.

One of the main research gaps concerns the strength-related behavior of fractal-inspired architectures. Although multiscale self-similar geometries are often associated with improved stiffness-to-weight or the strength-to-weight ratios, the way these structures respond to increasing load, local damage, and progressive failure is still not well established. Minor defects or stress concentrations at lower structural scales may propagate across hierarchical levels and trigger complex failure scenarios that cannot be captured adequately by simplified homogenized descriptions [88]. Future investigations should therefore focus on how hierarchical geometry affects local stress amplification, load redistribution, damage initiation, and crack propagation across scales, since such studies are necessary to determine whether fractal-inspired architectures can provide not only attractive effective responses, but also reliable load-bearing performance under realistic service conditions.

A second challenge involves deformation behavior. In fractal and hierarchical metamaterials, the overall response is often controlled by coupled mechanisms that emerge across different length scales. Local bending, buckling, rotation, and non-affine deformation modes may interact in a highly nonlinear manner, making it difficult to predict global structural behavior from conventional continuum-based models [83,88]. This creates a need for more spatially resolved approaches capable of tracking internal stress–strain evolution under realistic loading conditions. Because mechanical coupling occurs across multiple scales, the macroscopic response of fractal-inspired metamaterials cannot be fully understood through global stress–strain curves alone; future work should investigate how deformation develops sequentially within the architecture and how instabilities interact between hierarchical levels.

Energy absorption represents another important but still incompletely understood aspect of fractal-inspired metamaterials. Existing studies suggest that hierarchical architectures may enable distributed dissipation and delay catastrophic localization; for example, increasing the fractal order and introducing wall-thickness gradients in fractal lattice designs has been shown to substantially enhance specific energy absorption relative to conventional honeycomb baselines [83]. However, the quantitative relationship between fractal geometry, deformation sequence, and absorbed energy distribution remains insufficiently clarified, and it is still unclear how architecture controls whether absorbed energy remains localized near the loaded surface or is redistributed through the full structural depth. Hierarchical lattice designs have likewise been reported to improve energy-absorption efficiency relative to single-scale cellular structures, suggesting that multiscale architecture is a promising but still underexploited lever for tailoring dissipation pathways [94]. Future studies should therefore examine how absorbed energy is localized or delocalized through the material thickness, how local architecture influences dissipation pathways, and how such behavior can be optimized for protective and impact-related applications.

### 7.2. Manufacturability and Technological Challenges

From a technological perspective, manufacturability and scalability remain major constraints. The complexity of fractal geometries often exceeds the practical capabilities of conventional production routes, while even advanced additive manufacturing methods remain limited by resolution, throughput, material compatibility, and cost [88,95]. As a result, many reported structures remain closer to proof-of-concept demonstrations than to truly deployable engineering materials. Bridging this lab-to-field gap will require closer integration between unit-cell design, process-aware modeling, and characterization protocols suited to industrial qualification standards, rather than treating manufacturability as a constraint addressed only after the architecture has been finalized [95]. Four challenges in particular currently limit the translation from laboratory demonstration to engineering deployment. First, large-scale preparation processes for hierarchical and fractal-like lattices remain constrained by build volume, build time, and resolution limits of additive manufacturing platforms, so that geometries validated at coupon scale are not yet routinely reproduced at component scale with consistent quality [35,40,47,95]. Second, multiscale simulation of these architectures carries a substantial computational cost, since resolving deformation and failure at the finest hierarchical level while retaining a component-scale model typically requires either prohibitively fine meshes or homogenization schemes that can obscure the very scale-coupling effects the architecture was designed to exploit. Third, the mechanics of complex, multi-level configurations under combined or non-proportional loading (multiaxial, dynamic, cyclic) remains analytically and numerically difficult to solve in closed form, forcing continued reliance on case-specific finite-element studies rather than general design rules. Fourth, structural-topology optimization methods that perform well for single-scale lattices generalize poorly to hierarchical or fractal-like configuration spaces, where the design variables at different levels interact nonlinearly and the optimizer can converge to geometrically complex solutions whose manufacturability or robustness has not been verified [96].

### 7.3. Toward Integrated Theoretical, Numerical, and Experimental Validation

A further research gap lies in the limited integration of theoretical, numerical, experimental, and increasingly, data-driven approaches. Many studies focus on only one of these aspects, leading to fragmented understanding and reduced comparability between results. Machine-learning and other data-driven methods offer a complementary route for navigating the very large design spaces associated with fractal and hierarchical architectures, but their adoption in this specific subfield is still limited by data scarcity, restricted generalizability across geometries and loading conditions, and limited interpretability of learned structure–property relationships [96]. More unified research strategies, combining physics-based models with data-driven surrogates validated against experiments, are therefore required to establish robust relationships between hierarchical architecture and mechanical performance.

Taken together, these limitations indicate that the field still lacks a sufficiently mature mechanics-centered framework capable of connecting geometry, internal field evolution, and structural performance in a predictive manner. Addressing this gap is essential for transforming fractal-inspired metamaterials from conceptually appealing architectures into reliable engineering materials.

**Figure 10:**
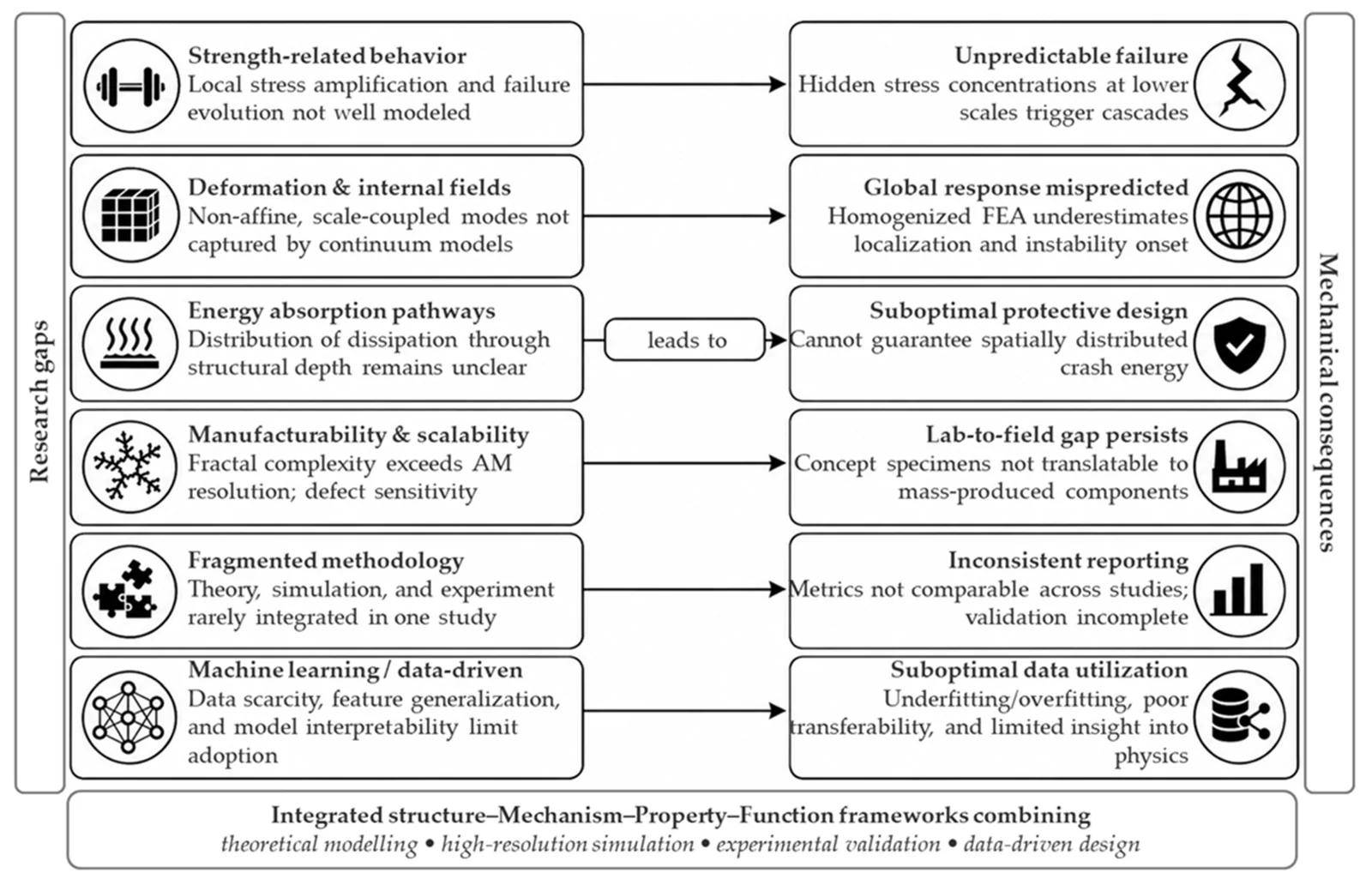
Research gaps and engineering implications in fractal and hierarchical metamaterials. The proposed structure-mechanism-property-function (structure-mechanism-property-function) framework is presented as an integrated strategy to address the identified challenges. The gaps are synthesized from limitations reported in [22,24,26,35,36,40,47,95,96]. The figure was created with assistance from ChatGPT (OpenAI) GPT-5.6 Luna and subsequently reviewed, edited, and scientifically validated by the authors.

The relationship between identified research gaps and their practical mechanical consequences is summarized in Figure 10. Each gap limits the field’s ability to design, predict, or validate fractal metamaterial performance under realistic conditions. Addressing these gaps requires integrated research strategies rather than isolated theoretical, numerical, or experimental studies. The key features and mechanical implications of fractal and hierarchical architectures are further consolidated in Table 3.

Table 3 highlights several directions in which future mechanics-oriented research on fractal-inspired metamaterials should be developed. Future work on fractal and hierarchical mechanical metamaterials should move beyond the description of unusual effective properties and toward a deeper mechanics-based understanding of how these properties emerge, evolve, and ultimately fail under loading.

Another important direction concerns the integration of fractal-inspired architectures into composite and multifunctional systems. Although the geometry of fractal-inspired metamaterials offers substantial mechanical potential, practical implementation requires better understanding of interface behavior, manufacturing tolerances, and structural reliability when these architectures are combined with different constituent materials or layered systems [83,95].

Beyond purely mechanical performance, the geometric complexity of fractal structures may also influence transport-related properties. In particular, future studies may explore whether multiscale connectivity and increased internal surface complexity can enhance thermal transport or heat dissipation under certain conditions. While this area remains less developed than the mechanical domain, it represents a promising extension of current research and may open new pathways for multifunctional material design.

Overall, future progress in this field will depend on the combined use of theoretical modeling, high-resolution numerical simulation, data-driven design, and experimental validation [96]. Establishing direct links between hierarchical geometry and strength, deformation, energy absorption, manufacturability, and possibly thermal transport will be essential for the rational design of next-generation fractal-inspired metamaterials, and for closing the gap between conceptually appealing architectures and reliable, manufacturable engineering materials.

### 7.4. Emerging Directions: Reverse Design, AI-Driven Topology Generation, and Manufacturing-Aware Complexity

Beyond the integration challenge discussed above, three closely related directions are attracting rapidly growing attention and merit explicit comment. Reverse (inverse) design methods specify a target mechanical response—a stiffness tensor, an energy absorption profile, or a band-gap frequency range—and search the geometric design space for a hierarchical or fractal-inspired architecture that achieves it, inverting the conventional forward analyze-then-iterate workflow used throughout most of the studies reviewed here [96]. Machine-learning-assisted and generative (AI) topology generation methods accelerate this search by learning structure-property mappings from simulation or experimental datasets. They inherit the multiscale generalization problem noted in Section 7.2: a model trained on single-scale lattice data does not automatically generalize to hierarchical configuration spaces, and generated geometries require the same manufacturability and experimental validation as conventionally designed ones [96]. Additive-manufacturing-process matching—co-designing the architecture together with the constraints of the intended printing process (resolution, self-supporting angles, powder or resin removal from internal cavities)—is increasingly treated as a first-class design variable rather than a downstream manufacturing check [35,40,47,86,90,91,92]. Taken together, these directions raise a question that recurs throughout this review: when is added structural complexity actually justified? Based on the evidence synthesized here, this review proposes that a hierarchical or fractal-like feature is mechanically meaningful—rather than complexity for its own sake—only when it satisfies at least three conditions: (i) it is assigned a distinct mechanical role not already performed by another scale (Section 3.4 and Section 4.1); (ii) its effect on strength, stiffness, or energy absorption has been demonstrated experimentally or numerically against a same-density single-scale baseline, not merely asserted; and (iii) it remains within the manufacturable and inspectable feature-size range of the intended production route (Section 7.2). Configurations that cannot satisfy all three criteria should, on the evidence reviewed here, be treated as unvalidated design proposals rather than as demonstrated performance gains.

## 8. Conclusions

This review has examined hierarchical and fractal-inspired mechanical metamaterials from a mechanics-oriented perspective, focusing on how multiscale architecture governs strength, deformation behavior, failure evolution, and energy absorption. The main conclusion is that such materials should not be interpreted as mechanically superior solely because they possess complex, periodic, hierarchical, or self-similar geometries. Their performance depends on whether the internal architecture performs a clear mechanical function: redirecting load paths, redistributing stress, activating favorable deformation modes, delaying localization, stabilizing post-buckling response, or enabling controlled energy dissipation. The specific contribution of this review, distinct from prior surveys of architected or auxetic metamaterials, is the explicit mechanics-oriented classification developed in Section 3, Section 4, Section 5 and Section 6, which organizes architectures by the deformation mechanisms they activate rather than by geometric appearance, together with the critical delineation in Section 6.2 of where fractal mathematical descriptors are mechanically meaningful and where hierarchical architectures are better described as fractal-inspired rather than fractal in the strict mathematical sense.

A recurring finding across the reviewed literature is that relative density alone is an insufficient descriptor of mechanical performance. Structures with the same density may exhibit fundamentally different stiffness, strength, recoverability, and failure behavior depending on whether deformation is governed by bending, stretching, membrane action, shell buckling, plasticity, local fracture, or hierarchical collapse. Stretch-dominated lattices improve load transfer compared with conventional bending-dominated cellular solids, but they remain sensitive to node geometry, imperfections, boundary conditions, and buckling. Shell-, plate-, and surface-based architectures can engage more material in load bearing through membrane stresses, but their practical performance depends strongly on imperfection sensitivity, post-buckling stability, and manufacturability. Nanolattices and ceramic metamaterials further demonstrate that architecture and size effects can modify apparent strength and recoverability, but these effects must be interpreted cautiously when moving from small specimens to engineering-scale components.

Hierarchy and fractality provide a broader design space by distributing mechanical function across multiple length scales. However, this review shows that hierarchy is beneficial only when each scale contributes to a specific mechanical role. Additional structural levels may improve stress redistribution, damage tolerance, or energy absorption, but they may also introduce stress concentrations, excessive compliance, weak junctions, or greater sensitivity to defects. Similarly, fractal geometry should not be treated as a universal route to higher stiffness or strength. Its value depends on the intended function and on the deformation mechanisms it promotes. In some applications, fractal or recursive layouts may be useful because they delocalize strain and increase compliance; in others, they may be advantageous because they coordinate progressive collapse, distribute energy dissipation, or influence dynamic response.

Instability-driven and programmable metamaterials further demonstrate that mechanical performance cannot be judged by peak stiffness or strength alone. Buckling, snap-through, and plasticity may act either as failure mechanisms or as functional design tools, depending on whether they are spatially controlled, reversible, dissipative, or stable after activation. This creates a shift from evaluating metamaterials as static points on stiffness–density or strength–density charts toward evaluating them as path-dependent structural systems. For this reason, future assessment should include post-buckling response, recoverability, residual deformation, cyclic durability, imperfection sensitivity, and failure localization, rather than relying only on initial elastic properties.

The automotive crashworthiness case study illustrates these principles in an application-driven context. Metamaterial and auxetic architectures can improve energy absorption, stabilize collapse, reduce mass, and contribute to multifunctionality. However, high specific energy absorption alone is not sufficient for crashworthiness. A useful crash absorber must also manage peak crush force, mean crush force, crush force efficiency, deformation stability, intrusion, and protected-component integrity. The reviewed studies show that honeycomb, lattice, auxetic, hierarchical, fractal, and additively manufactured structures may each be advantageous under different design objectives, but no single architecture is universally optimal. This reinforces the need for application-specific evaluation frameworks and standardized reporting of crashworthiness metrics.

Fractal theory also provides useful tools for understanding complex mechanical phenomena such as fatigue, fracture, vibration, surface interaction, and multiscale damage evolution. Its value lies in the ability to quantify irregular, scale-dependent, and self-similar features that are not adequately captured by classical Euclidean or homogenized descriptions. Nevertheless, the integration of fractal theory into mechanical metamaterial design remains incomplete. A stronger connection is still needed between fractal descriptors, internal stress–strain fields, deformation sequences, damage initiation, and experimentally measurable structural performance.

Several challenges continue to limit the transition of fractal and hierarchical metamaterials from promising laboratory demonstrations to reliable engineering materials. These include manufacturing resolution, scalability, material compatibility, imperfection tolerance, reproducibility, incomplete experimental validation, limited testing under dynamic and multiaxial loading, and inconsistent reporting of mechanical metrics. Many current studies remain focused on idealized geometries or small specimens, whereas practical applications require robust performance under realistic service conditions. Therefore, manufacturability, defects, and scale-up should be treated not as secondary implementation issues, but as central components of the mechanical design problem.

Future research should move toward integrated structure–mechanism–property–function frameworks. Such frameworks should combine theoretical modeling, high-resolution numerical simulation, experimental validation, and data-driven design to connect architectural parameters with internal field evolution and macroscopic performance. Particular attention should be given to strength-related behavior, progressive failure, deformation stability, energy absorption pathways, interface behavior in composite systems, and multifunctional responses such as vibration attenuation or thermal transport. The most promising direction is not the search for a single superior architecture, but the rational integration of complementary mechanisms across scales. In this sense, fractal and hierarchical mechanical metamaterials offer a powerful route toward next-generation architected materials, provided that their complexity is mechanically purposeful, experimentally validated, and compatible with scalable manufacturing.

## Figures and Tables

**Table 1:** Literature-based conceptual framework summarizing the relationships between crashworthiness metrics and structural design parameters *.

Architecture/Absorber Concept	Effect on Crashworthiness Metrics **					
	TEA	SEA	MCF	PCF	CFE	Intrusion Control
Conventional honeycomb	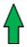	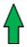	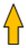	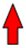	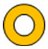	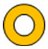
Hierarchical honeycomb	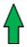	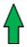 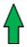	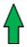	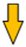	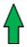	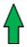
Auxetic lattice (re-entrant/chiral-derived)	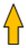	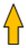	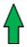	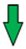	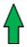	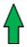
Hybrid honeycomb–auxetic	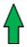	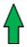	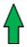 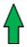	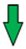	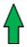 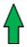	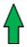
Cubic lattice-filled shell	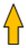	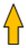	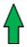	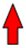	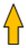	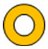
BCC lattice-filled shell	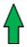 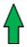	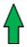 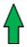	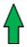 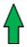	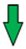	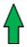 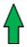	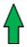 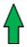

* The entries summarize qualitative trends reported across the cited crashworthiness literature [23,24,25,26,67,68,69,70,71,72,73,74,75,76,77,78,79,80,81,82] rather than results from a single controlled experimental comparison. Quantitative values depend on specific geometry, material properties, and loading conditions. ** TEA—total energy absorption; SEA—specific energy absorption; MCF—mean crush force; PCF—peak crush force (desirable); CFE—crush force efficiency (MCF/PCF). 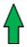

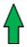
—strong improvement; 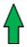
—improvement; 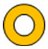
—neutral (no change); 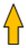/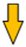
—minor improvement/deterioration; 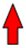
—deterioration; 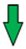
—reduction (desirable for PCF).

**Table 2:** Comparative overview of representative metamaterial architecture classes, deformation mechanisms, scaling behavior, failure modes, manufacturability, and application domains.

Architecture	Dominant Mechanism	Scaling Exponent *n*	Typical Failure Mode	Advantages	Limitations	Manufacturability/Applications
Stochastic/open-cell foam	Bending-dominated	*n* ≈ 2	Progressive cell-wall bending collapse	Low cost, simple processing	Poor stiffness/strength at low density [16,37]	Packaging, thermal/acoustic insulation
Octet/stretch-dominated truss	Axial stretching	*n* ≈ 1	Strut buckling or fracture at nodes	High stiffness-to-weight [36,39]	Sensitive to node imperfections and material defects [35,40]	additively manufactured lattices, lightweight load-bearing panels
Re-entrant/auxetic lattice	Rotating hinge-like flexure	*n* ≈ 1–2 (direction-dependent)	Ligament buckling, hinge fatigue	Negative Poisson’s ratio, indentation/impact resistance [17,34,38]	Anisotropic; direction-dependent properties	Crash boxes, protective padding, sensors
Shell/TPMS (gyroid, diamond, primitive)	Membrane (shell) stretching	*n* ≈ 1–1.5	Local shell buckling/wrinkling [18,19,35,40,44]	Smooth, imperfection-tolerant load paths; high SEA	Imperfection- and curvature-sensitive; complex tooling	Biomedical scaffolds, heat exchangers, crash absorbers
Hierarchical honeycomb (order 1–2)	Bending at multiple scales, delayed wall buckling	*n* varies by order (typically increases toward stretch-dominated with added hierarchy) [17,38,57]	Progressive, multi-scale plastic hinge formation	Extended stable crushing plateau; up to ~148% SEA gain reported [26]	Higher geometric/manufacturing sensitivity [24,26]	Crash boxes, hierarchical bumper fillers
Nanolattice (hollow/solid strut, micro/nano scale)	Size-dependent axial/shell deformation	*n* ≈ 1–1.7 (size-dependent)	Strut fracture or wall buckling; flaw-sensitive at larger scale [21,22,35]	Near-theoretical strength at small scale [22]	Scale-up to macroscopic components largely unproven [22,35,40,47]	Demonstrator-scale lightweight/ceramic components
Fractal-cut/recursive lattice	Scale-coupled bending–stretching (level-dependent)	*n* varies with iteration order and scale ratio	Damage delocalization or, if poorly designed, hidden stress concentrations [83]	Tunable stiffness/energy absorption via iteration order [83]	Design space not yet systematically mapped; limited experimental validation	Emerging: energy-absorbing panels, vibration bandgap structures

Note: The characteristics summarized in this table represent qualitative trends synthesized from the cited literature and should not be interpreted as results from a single controlled experimental comparison. Approximate stiffness scaling exponents are based on classical cellular-solids and lattice-mechanics analyses.

**Table 3:** Main mechanical implications of fractal and hierarchical architectures in metamaterials. Implications are a qualitative synthesis of the mechanisms discussed in Section 3, Section 4, Section 5 and Section 6 [16,17,18,19,20,21,22,23,24,25,26,27,28,29,30,31,32,33,34,35,36,37,38,39,40,41,42,43,44,46,47,48,49,50,51,52,53,54,55,56,57,58,59,60,61,62,63,64,65,66,83,84,85,86,87,88,89,90,91,92,93,94,95,96] rather than measured values for a specific architecture.

Fractal/Hierarchical Feature	Main Mechanical Implication	Strength-Related Relevance	Deformation-Related Relevance
Self-similar geometry across multiple scales	Enables coupling between local and global response	May improve stress redistribution and strength-to-weight efficiency	Activates scale-dependent deformation modes
Hierarchical load paths	Redistributes stresses through nested structural elements	May reduce local stress peaks and delay failure initiation	Promotes progressive rather than abrupt deformation
Increased geometric complexity	Expands the design space beyond conventional periodic lattices	May improve mechanical tailoring, but may also generate hidden stress concentrations	Leads to highly nonlinear and non-affine deformation behavior
Multiscale instability mechanisms	Allows local buckling, bending, and rotation to interact across levels	May either delay or accelerate failure depending on architecture	Strongly affects deformation sequence and global response
Distributed internal interfaces and branching motifs	Alters stress transfer and damage propagation pathways	Can improve load distribution if interfaces remain mechanically robust	Influences strain localization and deformation heterogeneity
High internal surface complexity	Increases opportunities for multifunctionality	Mechanical significance depends on load path continuity	May affect local compliance and structural adaptability
Additive-manufacturing compatibility of architected fractal-inspired systems	Enables realization of complex geometries not feasible by conventional routes	Supports experimental assessment of strength-related concepts	Allows controlled study of multiscale deformation patterns

## Data Availability

No new data were created or analyzed in this study. Data sharing is not applicable to this article.
